# The two-stage molecular scenery of SARS-CoV-2 infection with implications to disease severity: An in-silico quest

**DOI:** 10.3389/fimmu.2023.1251067

**Published:** 2023-11-21

**Authors:** George Potamias, Polymnia Gkoublia, Alexandros Kanterakis

**Affiliations:** ^1^Computational Biomedicine Laboratory (CBML), Institute of Computer Science, Foundation for Research and Technology-Hellas (FORTH), Heraklion, Greece; ^2^Graduate Bioinformatics Program, School of Medicine, University of Crete, Heraklion, Greece

**Keywords:** SARS-CoV-2, COVID-19, differential expression analysis, pathway enrichment analysis, diagnostic and prognostic classifier models, machine learning

## Abstract

**Introduction:**

The two-stage molecular profile of the progression of SARS-CoV-2 (SCOV2) infection is explored in terms of five key biological/clinical questions: (a) does SCOV2 exhibits a two-stage infection profile? (b) SARS-CoV-1 (SCOV1) vs. SCOV2: do they differ? (c) does and how SCOV2 differs from Influenza/INFL infection? (d) does low viral-load and (e) does COVID-19 early host response relate to the two-stage SCOV2 infection profile? We provide positive answers to the above questions by analyzing the time-series gene-expression profiles of preserved cell-lines infected with SCOV1/2 or, the gene-expression profiles of infected individuals with different viral-loads levels and different host-response phenotypes.

**Methods:**

Our analytical methodology follows an in-silico quest organized around an elaborate multi-step analysis pipeline including: (a) utilization of fifteen gene-expression datasets from NCBI’s gene expression omnibus/GEO repository; (b) thorough designation of SCOV1/2 and INFL progression stages and COVID-19 phenotypes; (c) identification of differentially expressed genes (DEGs) and enriched biological processes and pathways that contrast and differentiate between different infection stages and phenotypes; (d) employment of a graph-based clustering process for the induction of coherent groups of networked genes as the representative core molecular fingerprints that characterize the different SCOV2 progression stages and the different COVID-19 phenotypes. In addition, relying on a sensibly selected set of induced fingerprint genes and following a Machine Learning approach, we devised and assessed the performance of different classifier models for the differentiation of acute respiratory illness/ARI caused by SCOV2 or other infections (diagnostic classifiers), as well as for the prediction of COVID-19 disease severity (prognostic classifiers), with quite encouraging results.

**Results:**

The central finding of our experiments demonstrates the down-regulation of type-I interferon genes (IFN-1), interferon induced genes (ISGs) and fundamental innate immune and defense biological processes and molecular pathways during the early SCOV2 infection stages, with the inverse to hold during the later ones. It is highlighted that upregulation of these genes and pathways early after infection may prove beneficial in preventing subsequent uncontrolled hyperinflammatory and potentially lethal events.

**Discussion:**

The basic aim of our study was to utilize in an intuitive, efficient and productive way the most relevant and state-of-the-art bioinformatics methods to reveal the core molecular mechanisms which govern the progression of SCOV2 infection and the different COVID-19 phenotypes.

## 1 Introduction

On January 10, 2020 WHO declared the outbreak of 2019-nCoV, which was the first name assigned to the new disease. On February 11, 2020 WHO named the disease as COVID-19 (COronaVIrus Disease 2019). On the same date the International Committee on Taxonomy of Viruses (ICTV) announced “severe acute respiratory syndrome coronavirus 2 (SARS-CoV-2)” as the name of the causative agent for the disease. WHO declared the outbreak a ′Public Health Emergency of International Concern′ on January 30, 2020, and a pandemic on March 11, 2020. From the beginning of the pandemic till today (mid-June, 2023) nearly 768M infected cases and a total of about 6.95M deaths have been reported worldwide (data from the WHO dashboard, covid19.who.int), of course, with diverging figures across different continents, regions and nations. The prevalence figures for the disease are still under exploration, with modelling studies to report estimates with huge divergences between them, from a low 1.5% (USA, serum study, April 2020) to a high 13.7% (USA, PCR study, March 2020) (1). In addition, virus variants as well as regional and workplace characteristics strongly influences the respective estimates (2). The reported rates for asymptomatic cases are more indicative, and concern mainly cases with no clinical symptoms at the time tested positive. According to the results of two meta-analysis studies (3, 4), nearly 35-41% of confirmed cases do not develop noticeable symptoms, and this has been attributed as the main cause of the disease widespread (5, 6). As COVID-19 pandemic is still in progress the figures regarding COVID-19 mortality rates remain still vague. Of interest is the comparison between the mortality rates of all different types of the Influenza (INFL) infection and COVID-19. According to WHO, INFL results in 3-5 million serious cases worldwide every year, with about 300,000 - 650,000 deaths attributed to the disease. The majority of INFL infected people do not seek for medical attention, and so, the actual INFL cases every year are estimated to be about 100 times higher, which give us a raw estimate of about 4 billion incidents. Under this assumption, a crude estimate for INFL infection fatality rate (IFR) is about 0.1%. This figure is substantially lower compared to respective COVID-19 IFR estimates which, according to a recent model-based study regarding the first pandemic wave, ranges from 0.15–0.43% in low-income to 0.79–1.82% in high-income countries with the differences to reflect the older demography of high-income countries (7). In a systematic model-based (a Bayesian framework is utilized) meta-analysis that reviewed 3.012 age-specific seroprevalence studies across 53 counties, the authors report a decrease in the median IFR, from nearly 0.47% on April 2020 to about 0.31% on January 2021 (8). In an enlightening paper, about one year after the pandemic was declared (9), some interesting results and estimates are reported regarding the immunological characteristics of COVID-19 and its putative transition to an endemic state. The authors postulate three rational assumptions that support their hypotheses and estimates: (a) faster transmission results in a quicker transition to the endemic state but with increased mortality; (b) social distancing saves lives, delays endemicity and buys crucial time for vaccine roll-out, and (c) vaccination speeds up the transition to the endemic state and reduces the death toll. Their modelling framework provides forecasts about the progress of COVID-19 mortality figures in a time scale of 2.5 to 10 years (considering different disease reproduction numbers/R_0_). In addition, the authors provide a very interesting figure that summarizes their forecast. It states that COVID-19 will reach an IFR of about 0.1% (the actual IFNL rate) about three years after the pandemic emergence, and even less in subsequent years. Of course, for this to happen the virus must spread to about 99% of the general population, either through childhood infections (with no or low symptomatology) and/or through mass vaccination programs^1^. The forecast seems to be confirmed following the recent spread of the more ′relaxing’ Omicron SARS-CoV-2 variants and the roll-out of mass vaccination programs worldwide.

Despite the fact that SARS-CoV-2 (henceforth as SCOV2) became a pandemic, it is actually the third serious outbreak caused by Coronaviruses in the last 20 years. The first SARS-CoV (henceforth as SCOV1) outbreak happened in Hong Gong around 2002–2003 (10), and MERS-CoV (the 2nd SARS) at Saudi Arabia and Jordan in 2012 (11, 12). The zoonotic origin of all three infections is presented as the most justified theory so far, with two scenarios proposed for their evolution and transfer to humans: (a) natural selection in an animal host before zoonotic transfer and (b) natural selection in humans following zoonotic transfer (13). A number of studies have demonstrated a strong similarity between the three infections in terms of their clinical manifestations (14, 15). In addition, it is well established that SCOV2 shares about 79+% and 50% of its genome with SCOV1 and MERS, respectively (16, 17). The close phylogeny between SCOV1 and SCOV2 lineages (both members of the sarbecovirus family) allows comparative studies between SCOV2 and SCOV1 infections.

The pathogenesis of SCOV2 infection is complex, with the exact reasons of its fatality still being explored. The fatal events of SCOV2 infection are linked with the so-called ′cytokine storm′ syndrome (CSS) (18) that may also occur in other viral infections (e.g., Ebola virus, Dengue virus, INFL/H1N1, SCOV1 and MERS-CoV). Cytokines include interferons, tumor necrosis factors (TNFs), interleukins, and chemokines that regulate host defense responses and play an important role in mediating innate immune responses, mainly by regulating inflammatory reactions, excessive chronic production of which promote inflammatory diseases. CSS plays a decisive role in the progression of SCOV2 infection and is an important factor for severe and fatal outcomes (R. 19). Studies have shown that COVID-19 patients with severe symptoms exhibit much higher levels of white blood cells, neutrophils, procalcitonin and other inflammatory markers compared to patients with mild symptoms (20). It is postulated that CSS presents a systemic inflammatory response to the infection, which leads to over-activation of immune cells and to uncontrolled production of inflammatory cytokines (21). In a recent study that compared the immune profiles between patients with severe respiratory INFL illness and moderate/severe COVID-19 patients, CSS was found to be relatively rare among moderate and severe COVID-19 infections, with most COVID-19 patients to exhibit suppressed immune profiles, mainly noticeable in IFN signaling, relative to those detected in INFL patients (22). This is in contrast to the findings and assumptions dominating the pertinent literature (23), and were challenged by other clinical meta-analysis studies (24). So, a question of major importance relates to the staging of SCOV2, i.e., the progression from an early to a later infection stage and especially, to the molecular and regulatory machinery that underlies and characterizes this progression.

### 1.1 SCOV2 molecular infection profile: An impaired resistance-tolerance interplay

The two-stage progress of SCOV2 infection is directly linked to the suppression of key host immune actions, with various studies showing that SCOV2 causes the ′blocking′ of type I interferons (IFN-I) and the suppression of immune/defense responses in the first hours and days of infection (25). The aberrant functioning of fundamental innate immune responses during the early infection stage leads to uncontrolled virus replication, and to subsequent excessive leukocyte recruitment and uncontrolled inflammatory events at the later infection stages (26). IFN-Is are pleiotropic cytokines composed by a family of IFN proteins which are encoded by at least thirteen IFN-related genes (27). Contrasted to other virus infections, the early stages of SCOV2 infection is carved by reduced IFN-I/III responses with parallel induction of proinflammatory chemokines (28). In a study that included patients with fatal pneumonia caused by SCOV2, a two-stage disease evolution is signified and linked to respective viral-load levels (29). The two-stage SCOV2 progression profile is also highlighted in a recent single-cell RNAseq study contrasting the infection profiles between children and adults, demonstrating that the pre-activation of key interferon-stimulated genes (ISGs) in the upper airways controls early SCOV2 infection in children (30). A key to unlock and characterize the transition from suppressed immune responses at the early stages of the infection to their over-activation at later stages may be obtained from the interplay between two fundamental host defense strategies, namely: *resistance* and *tolerance* (31). Disease resistance engages various physiological and molecular host immunity processes, both innate and adaptive, aiming to reduce the pathogen’s load. IFN-signaling, with its crosstalk with apoptotic, inflammation and cell stress-response pathways, hold the primary role for these processes to be triggered and elicit a host antiviral state (32). Disease tolerance triggers host responses aimed to contain the damage to the affected tissue and support its function by tolerating the pathogen’s burden. In the upper respiratory tract/URT, the initial entry and replication site for SCOV2, tolerance defense mechanisms aim to sustain the exchange of gas and blood oxygenation. When resistance is weakened and the virus spreads to the lower respiratory tract, tolerant defense processes are triggered in order to preserve the alveolar structures which are crucial for gas exchange. But, the same IFN-I genes that guide the antiviral activities poses a ′dual-role′, as they are engaged in the modulation of destructive immune responses that cause tissue damage (33). So, down-regulation of IFN-Is/ISGs during the early infection stages and their exaggerated induction during the later stages may be proved harmful for the smooth function of the needed tolerance processes, leading to severe, and putatively fatal, clinical outcomes. It is indicative that for COVID-19 severe cases with prolonged hospitalization, fatal events mostly occur after the virus is cleared, a fact that designates the continuation of host resistance mechanisms even if the threat is eliminated (34).

### 1.2 The molecular canvas of immune and defense response during the infection course: the SCOV2 case

**Figure 1:**
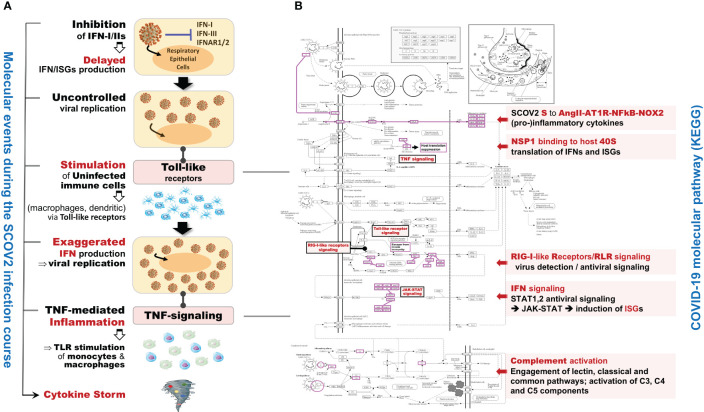
**(A)** The main molecular processes during SCOV2 infection [inspired and fully redesigned from (52)]. **(B)** Annotated COVID-19 KEGG pathway (www.genome.jp/pathway/hsa05171); key regulatory subnetworks and engaged pathways are highlighted.

In the initial phase of (viral or bacterial) infection the first task for the host cells is to identify virus invasion via specialized pattern recognition receptors (PRRs) and the recognition of particular pathogen-associated molecular patterns (PAMPs) (35). These molecules are sensed by specific receptors and the triggering of dedicated molecular pathways (e.g., Toll-like and RIG-I). Following this course, various host defense processes are activated including, direct antiviral molecules and inflammatory mediators such as IFN-I/III, tumor necrosis factor (TNF), interleukin 6 (IL-6) and other chemokines. Their role is to trigger a series of molecular processes that lead to the induction of ISGs and various proinflammatory cytokines (32, 36, 37). The main role of ISGs is to block virus replication, with several negative IFN regulators to also target PRRs in order to reduce the magnitude of host responses. The complex canvas of IFN/ISGs during the full life-cycle of various viral infections, from their attachment to their maturation, is exemplified in (38). The authors present a total of 24 genes and highlight their specific key antiviral roles and activities, namely: CH25H − affects virus entry at the host-membrane fusion events; IFITM1/2/3/5 − inhibit endocytic-fusion events; TRIM5 − prevents uncoating of viral RNA; MX1/2 family − block endocytic traffic of virus particles; OAS1/2/3/L, RNaseL, MOV10 and ZAP − degrade and block translation of viral mRNAs; IFIT1/2/3/4/5 − inhibit viral protein translation; TRIM22 − inhibit viral transcription as well as the replication and trafficking of viral proteins to the plasma membrane; ISG15 − inhibit viral translation, replication and egress; RSAD2 (Viperin) − inhibit viral replication at the plasma membrane; BST2 (Tetherin) − trap the escaped mature virus particles on the plasma membrane and inhibit viral release. Despite this, SCOV2 has employed several different mechanisms to escape the host immune/defense processes, mainly via the ‘blocking’ of IFN-signaling and induction of ISGs. This leads to productive viral replication during the early stages of infection, a situation that greatly contributes to COVID-19 pathology and severity (39, 40). It is well-established that not only SCOV2 but also other viral infections interfere with interferon signaling (39, 41) but, in contrast to other respiratory viral infections, COVID-19 patients demonstrate downregulation of interferon signaling pathways at the early infection stages (22, 42, 43). The crucial role of dendritic cells (DCs) as the medium that bridges innate and adaptive immunity is also signified, as DCs have a decisive role for the activation of antiviral T-cells. They possess the unique ability to present, through the major histocompatibility complex class I/MHC-I, cell-associated antigens to CD8+ cytotoxic T-cells, with IFN-Is to act as enhancers that promote DC maturation (44, 45). It is shown that reduced production of IFN-Is in DCs during the early infection stages leads to aberrant T-cell responses, a fact identified as one of the causes for severe SCOV2 infection outcomes (46). Furthermore, in a bioinformatics analysis that relates DCs with immune-induced diseases, a set of ten key genes are reported to play a decisive role, namely: ISG20, IFITM1, HLA-F, IRF1, USP18, IFI44L, GBP1, IFI35, IFI27 and IFI6/IFI27-like protein/ISG12 (47). There is also strong supportive evidence that SCOV2 activates the complement, mainly through direct recognition of SCOV2’s spike S and nucleoplasmid N proteins, leading to activation of various complement pathways and triggering of C3, C4 and C5 components (48), with the subsequent direction of IgG and IgM antibodies against the receptor-binding domains of virus proteins (49, 50). The crucial role of the virus non-structural protein 1 (NSP1) in shutting down mRNA translation of IFNs, proinflammatory cytokines and ISGs, through its binding to the host 40S ribosomal subunit, has also been highlighted. This places NSP1 as one of the main immune evasion factors of SCOV2, mainly through suppression of RIG-I/DDX58 virus sensing proteins (51). As a guide to the aforementioned observations, **Figure 1** outlines the main molecular events during the full SCOV2 viral life-cycle (left part of the figure). At the right part of the figure the KEGG COVID-19 disease pathway is shown, with the involved key regulatory sub-networks highlighted in pink color. The annotations for the key molecular processes taking place are shown in light-pink boxes. The engaged signaling pathways (signaling of TNF, Toll-like receptors, RIG-I receptors and JAK-STAT/IFN-signaling) are also indicated.

The aforementioned observations and discussion make clear that the elucidation of the molecular landscape underlying and governing SCOV2 infection should be explored and assessed in relation to the progression stages of the infection. To this end, a central element to our analysis methodology is the designation of SCOV2 infection stages from the gene-expression profiles of cell-line samples infected at different time-points. We conducted a series of experiments and respective in-silico bioinformatics analyses on a series of gene-expression datasets in order to explore a spectrum of critical biological and clinical questions related to SCOV2 infection, with the following targets: (i) establishment of a reliable methodology for the designation of SCOV2 infection stages (e.g., early, late) on the one hand, and the designation of host SCOV2 infection phenotypes on the other (e.g., low/high viral-load levels or early/late host infection responses) based on gene-expression (RNAseq or microarray) profiles; (ii) identification of differentially expressed genes (DEGs), enriched biological processes and pathways, as well as creation of coherently grouped networks of genes as representatives for the molecular fingerprints underlying SCOV2 infection; (iii) explore and reveal the common molecular fingerprints underlying both SCOV2 and SCOV1 infections, as well as respective fingerprints that differentiate between SCOV1/2 and INFL infections; (iv) identification of significant molecular imprints that distinguish between different SCOV2 viral-load levels and between different severity phenotypes (e.g., mild, moderate, severe/critical), and finally (v) induction and assessment of COVID-19 diagnostic and prognostic machine-learning (ML) models as an aid to support respective clinical decision-making processes. To succeed our targets, in the next section we detail on a carefully designed and documented bioinformatics pipeline that operates on a set of indicative public-domain gene-expression datasets in order to tackle a set of critical tasks that concerns SCOV2 infection.

## 2 Materials and methods

### 2.1 Computational framework

For our experiments and analyses we utilized the iDEP server (bioinformatics.sdstate.edu/idep). iDEP is an R-Shiny web-based application equipped with state-of-the-art bioinformatics techniques based on the smooth integration of respective R-packages (53), including: differential expression gene (DEG), exploratory (k-means clustering, principal components analysis/PCA) and enrichment/pathway analysis, (54, 55), as well as advanced visualization capabilities (heatmaps, hierarchical clustering trees, enriched pathway maps and gene networks). To further support our analyses, and beside the annotation services provided by iDEP, we utilized g:profiler (56) for the conversion of gene IDs between different gene-expression annotations (biit.cs.ut.ee/gprofiler/gost). A novel and fundamental component of our analytical methodology is the construction of coherent clusters of networked genes as the representative core molecular fingerprints that characterize SCOV2 infection stages and the different host phenotypes (i.e., the low/high viral-load levels and the early response to the infection). For this, we relied on the STRING server (string-db.org) for functional annotation, clustering, gene network construction and visualization of correlations and interactions between the induced DEGs. In section 2.1.2 we present in more detail the methods and techniques that were utilized in our experiments.

### 2.2 Tasks, experimental set-up and analysis pipeline

#### 2.2.1 Tasks, datasets and setup of experiments

Fifteen gene-expression datasets from NCBI’s gene expression omnibus/GEO repository (www.ncbi.nlm.nih.gov/geo) were used in order to address the aforementioned biological and clinical questions (**Table 1**). The whole quest is organized around six tasks that correspond to the raised biological/clinical questions. The datasets that were used in order to handle and cope with each task and conduct the respective experiments, accompanied by the respective data pre-processing details, are presented in the sequel. Recognizing the value of reproducible science, we provide at the end of the deposited **Supplement File** (Supplement.pdf) a table (**Supplementary Table 1**) that summarizes all the data-preprocessing details (e.g., sample/gene filtering and data normalization specifics), as well as, the specific parameterization setups for each of the performed experiments (e.g., false discovery rate/FDR and fold-change/FC cutoffs, the number of induced differential genes/DEGs and the number of fingerprint genes). To support the need of replication studies two additional files are also provided as **Supplementary Material**: (i) the compressed file Datasets.zip that contains all the processed datasets used in the conducted experiments, and (ii) the file DEGs.xlsx that lists the induced DEGs and fingerprint genes that were resulted from all the conducted experiments, accompanied by their unions and intersections being reported in the paper.

**Table 1:** Datasets used to address the posted biological/clinical questions and tackle the respective tasks (refer to section 2.1.1).

Dataset	Sample type	Platform	#	Task
GSE151513	cell-lines	RNAseq	1	The two-stage SCOV2 core molecular fingerprint
GSE158930				
GSE33267	cell-lines	Microarray	2	The common molecular fingerprint of SCOV2 and SCOV1 infections
GSE148729		RNAseq		
GSE47960	cell-lines	Microarray	3	Differentiation between SCOV1 and INFL/H1N1 molecular profiles
GSE152075	patient samples	RNAseq	4	Differentiation between the molecular fingerprints of low/high SCOV2 viral-load levels
GSE156063				
GSE166190	patient samples	RNAseq	5	The molecular fingerprints of early SCOV2 infection responders
GSE161731				
GSE156063 GSE188678 GSE163151	patient samples	RNAseq	6	Building and assessment of SCOV2 diagnostic classifier models
GSE152418 GSE178967 GSE172114 GSE177477	patient samples	RNAseq	6	Building and assessment of SCOV2 prognostic classifier models

##### 2.2.1.1 Task1: The two-stage SCOV2 core molecular fingerprint

To address this task we used two datasets, GSE151513 and GSE158930. GSE151513 comprises gene-expression profiles from Calu-3 human lung adenocarcinoma cell-lines infected with SCOV2 virus at a multiplicity of infection (MOI) of 2. Poly-A RNAseq gene-expression profiles were acquired with the ′Illumina HiSeq 2500 (Homo sapiens)′ platform (15,761 of them are included in the provided original dataset as gene-filtering was applied). The cell-line samples were collected at six hours post-infection (hpi) time-points: 0, 1, 2, 3, 6 and 12 hours, in triplicates, (n=18, three replicates for each infected sample). No normalization was performed as the original data were already normalized^2^. The results of the study are published in (57). We applied gene filtering on the available RNAseq data, removing 40% of gene-transcripts, ranked by their maximum expression level across all samples, leaving 9,457 gene-transcripts for further analysis. The results from the experiment conducted with the GSE151513 dataset are presented in section 3.1.1, and demonstrate a two-stage core molecular fingerprint that contrast between early and late SCOV2 infection stages. In order to further confirm our findings, we used the GSE158930 RNAseq dataset. GSE158930 comprises gene-expression profiles from ALI (air-liquid interface) cultures of primary human bronchial epithelial cells (HBECs) infected with SCOV2 virus at a MOI of 1. RNAseq gene-expression profiles were acquired with the ′Illumina NextSeq 500′ platform (57,905 Ensembl gene-transcripts). The cell-line samples were collected at five hpi time-points: 4, 24, 48, 72 and 96 hours (in du-/tri- or quadr-uplicates, n=19 infected samples). The results of the study are published in (58). We also applied gene filtering, removing gene-transcripts with CPM<5 across all samples, leaving 9,833 gene-transcripts for further analysis. In addition, sample filtering was also applied, keeping only the duplicate samples (i.e., replicates 1 and 2, as not all samples were profiled in tri- or quadru-plicates), leaving 10 infected samples to analyze. The filtered gene-expression counts were transformed using the variance stabilizing transformation (VST) method (59). The results from the experiment with the GSE158930 dataset are also presented in section 3.1.1.

##### 2.2.1.2 Task2: The common molecular fingerprint of SCOV2 and SCOV1 infections

To contrast the molecular profiles underlying SCOC2 and SCOV1 infections we used two datasets, GSE33267 and GSE148729. GSE33267 includes the gene-expression profiles of Calu-3 cell-line samples infected with the wild-type (icSARS) or the DORF6 (mutant not expression ORF6 protein) SCOV1 strains at a MOI of 5. The gene-expression profiles were acquired with the ′Agilent Whole Human Genome Microarray 4x44K G4112F (Feature Number version)′ microarray platform (33,631 gene-probes). Samples were collected in triplicates at eleven different hpi time-points, namely: 0, 3, 7, 12, 24, 30, 36, 48, 54, 60 and 72 hours. The results from this study are published in (60). No data normalization was performed as the original gene-expression profiles were already quantile normalized and log2 transformed. We retained just the icSARS/SCOV1 samples (n=33); the mock-treated and DORF6 samples were discarded. The feature numbered probes were mapped to human gene nomenclature committee/HGNC^3^ official gene symbols, relying on the respective iDEP’s mapping process. Gene filtering was also applied where, 40% of genes ranked by their maximum expression level across all samples were discarded, leaving 10,353 genes for further analysis. The results from the experiment conducted with the GSE33267 dataset are presented in section 3.1.2, and show a similar two-stage core molecular fingerprint underlying both SCOV2 and SCOV1 infections. Confirmation of the findings was done using the GSE148729 dataset. GSE148729 comprises RNAseq gene-expression profiles of Calu-3 human cell-line samples infected with SCOV1 (Frankfurt strain) and SCOV2 (patient isolate BetaCoV/Munich/BavPat1/2020| EPI_ISL_406862) viruses at a MOI of 0.33, using bulk and single-cell polyA-RNA, smallRNA, and totalRNA sequencing. In our experiments we focus on the total-RNAseq gene-expression data. Gene-expression profiles were acquired with the ′Illumina NextSeq 500/HiSeq 4000 (Homo sapiens)′ platform (40,648 Ensembl gene-transcripts) over three hpi time-points: 4, 12 and 24 hours, in duplicates (n=12, 6 samples for each virus infection). The results of this study are published in (61). Gene filtering was also applied where, gene-transcripts with CPM<5 across all samples were discarded, leaving 10,535 Ensembl gene-transcripts for further analysis, with their count values being VST transformed. The results from this experiment are also presented in section 3.1.2.

##### 2.2.1.3 Task 3: Differentiation between SCOV1 and INFL/H1N1 molecular profiles

To address this task, we used the GSE47960 dataset that is widely used in various relevant studies. It contains the gene-expression profiles of human airway epithelial (HAE) cell cultures infected with the wild-type (icSARS) SCOV1 strain (n=34), and the H1N1 influenza strain (n=20), with samples collected at nine different hpi time-points: 0, 12, 24, 36, 48, 60, 72, 84 and 96 hours for SCOV1, and seven hpi time-points: 0, 6, 12, 18, 24, 36 and 48 hours for H1N1. The larger extend of SCOV1 hpi time-points is expected, as the respective immune/defense responses, in contrast to INFL/H1N1, are delayed in SCOV1 infection. This is mainly due to the larger extent of the respective incubation periods; a mean of 2 days for the INFL/H1N1 2009 pandemic, 2-7 days for SCOV1 and 4-12 days for SCOV2 (62). Gene-expressions were profiled with the ′Affymetrix Human Gene 1.0 ST Array′ microarray platform (32,067 gene-probes). The results of the study are published in (63). In our experiment we kept just the samples infected with the icSARS/SCOV1 strain; the samples infected with other strains (SARS-dORF6 and SARS-BatSRBD) were discarded. No normalization was performed, as the gene-expression profiles in the original dataset were already quantile normalized, and no gene-filtering was applied. The results of this experiment are presented in section 3.1.3 and demonstrate a stronger, compared to SCOV1, immune/defense response profile during the early H1N1 infection stage.

##### 2.2.1.4 Task 4: Differentiation between the molecular fingerprints of low/high SCOV2 viral-load levels

To address this task we used two datasets, GSE152075 and GSE156063. GSE152075 comprises RNAseq profiles (acquired with the Illumina ′NextSeq 500′ gene-level platform; 35,784 genes) of nasopharyngeal (NP) swabs from 430 SCOV2 infected individuals and 54 negative controls. In our experiments we retained and focused just on the adult samples (age >= 20). SCOV2 viral-load were assessed by N1Ct, the PCR cycle threshold (Ct) of the SCOV2 nucleocapsid N1 target. As suggested in the original study publication (64), N1Ct values were discretized into three intervals that reflect respective viral-load levels: LOW (25.1 ≤ N1Ct ≤ 30.5, average N1Ct = 26.4, n=59) and HIGH (12.3 ≤ N1Ct ≤ 18.0, average N1Ct = 16.2, n=72); the samples with medium viral-load levels (18 ≤ N1Ct ≤ 25) were disregarded as we are interested to contrast between extreme viral-load levels. Gene filtering was applied where, genes with CPM<2 in at-least 59 samples (the number of samples with low viral-load) were filtered-out, leaving 8,130 genes for further analysis. The original gene read-counts were VST transformed. The results from the experiment with the GSE152075 dataset are presented in section 4.1, and indicate that low viral-load cases exhibit a suppressed immune response profile similar to the one underlying the early SCOV2 infection stage. Confirmation of findings was performed using the GSE156063 dataset. GSE156063 comprises RNAseq profiles acquired with the ′Illumina NovaSeq 6000′ ensemble gene-transcript level (15,979 Ensembl transcripts are included in the provided original dataset as gene-filtering was applied) of nasopharyngeal/oropharyngeal (NP/OP) swabs from 234 patients with acute respiratory illnesses (ARIs), infected either by SCOV2 or by some other virus or bacteria. The results of the study are published in (65). The infection type for each sample was acquired early in the infection stage by metagenomic sequencing (mNGS). mNGS integrates infection transcriptional signatures, and has proved its significance as a diagnostic tool for the assessment of ARIs, including those caused by SCOV2 infection (66–69). Viral-load is quantified in reads-per-million (RPM) values. In our experiments, only the samples with log_10_RPM>0 were retained for further analysis. The log_10_RPM values were quantile discretized into four nominal values, namely: too-high (average log_10_RPM=5.4, n=17), high-to-medium (4.3, n=16), low-to-medium (2.3, n=16), and too-low (1.15, n=17); samples with medium viral-loads (average log_10_RPM=3.0) were disregarded. Discretization was performed using scikitlearn’s KBinsDiscretizer python implementation following a quantile strategy. Too-high and high-to-medium samples were assigned to the HIGH viral-load class (3.75 ≤ log_10_RPM ≤ 5.89, average = 4.85, n=33), and the too-low and low-to-medium samples to the LOW class (0.14 ≤ log_10_RPM ≤ 2.55, average = 1.69, n=33), respectively. Gene filtering was additionally applied where, genes with CPM<5 over all samples were filtered-out, leaving 3,551 gene-transcripts for further analysis. The original gene read-counts were VST transformed. In both experiments we focused on the SCOV2 infected samples in order to differentiate between HIGH and LOW viral-load levels. The results from the experiment with the GSE156063 dataset are also presented in section 4.1.

##### 2.2.1.5 Task 5: The molecular fingerprints of early SCOV2 infection responders

To address this task we utilised two datasets, GSE166190 and GSE161731. GSE166190 includes RNAseq profiles of a total of 20 SCOV2 infected individuals, 11 adult (≥20 years old) and 9 children (≤16 years old). RNAseq profiles were acquired with the Illumina ′HighSeq 4000 (Homo sapiens)′ platform; 58,825 Ensembl gene-transcripts. For each individual the days post onset of symptoms/DPOS, as reported by each individual, were recorded and divided into five intervals: interval 1 (0-5 DPOS; the early infection stage), interval 2 (6-14 days), interval 3 (15-22 days), interval 4 (23-35 days), and interval 5 (36-81 days). The original publication of this study (70) report results that contrast between the response profiles of children and adults. Gene filtering was applied where, gene-transcripts with CPM<3 in at-least half of the samples were discarded, with the read-counts being VST transformed. From them, gene-transcripts with official HGNC gene symbols were retained for further analysis (13,027 unique genes). The results from the experiment with the GSE166190 dataset are presented in section 4.2, and show that early responders exhibit a robust antiviral immune response in the early stages of infection which, inverses the standard two-stage SCOV2 infection profile. To further confirm our findings, we used the GSE161731 dataset. GSE161731 includes RNAseq profiles of a total of 77 whole-blood samples from COVID-19 patients (the RNAseq gene-expression profiles were acquired via the ′Illumina NovaSeq 6000′ platform; 60,675 Ensembl transcripts). Whole blood samples were collected between 1-35 days post infection and divided based on disease severity and time from symptom onset. In particular, as reported in the original study publication (71), the individuals were assigned to three classes according to their reported time from onset of symptoms, early (≤10 days, the EARLY responders), intermediate (>10-21 days, the intermediate/MID responders) and late (>21 days, the LATE responders), with some of them being hospitalized. Each individual was sampled at different post infection time-points. For each of the multi-sampled individuals we kept just one sample, the one that corresponds to the individual’s earliest sampling. That is, if an individual is assigned to the early class (i.e., reported in its first hospital visit, ≤10 days from the onset of symptoms), and was then re-sampled at later time-points, the sample of the first hospital visit is retained. In this way we assure that the gene expression profile of each retained sample corresponds to an early, intermediate or late response of each individual. The samples were assigned to the following classes: EARLY_NO (not hospitalized early responders, n=9), EARLY_YES (hospitalized early responders, n=5), MID_NO (not hospitalized intermediate responders, n=19), MID_YES (hospitalized early responders, n=6) and LATE_NO (not hospitalized late responders, n=6). With this process 45 unique SCOV2 infected individuals were retained for further analysis. A new class variable is introduced, ′onset_hosp′, that holds the respective combined (days from symptoms onset and hospitalization status) nominal values. Gene filtering was also applied where, the transcripts with CPM<5 in at-least 10% of the retained samples, and which are mapped to official HGNC gene symbols, were retained for further analysis (11,497 unique genes), with the read-counts being VST transformed. The results from the experiment with the GSE161731 dataset are also presented in section 4.2.

##### 2.2.1.6 Task 6: Building and assessment of SCOV2 diagnostic and prognostic classifier models

The union of seven lists of genes being included in the core molecular fingerprints induced from specific experiments aforementioned above compose a set of 52 genes that were utilized for the devise and assessment of SCOV2 and COVID-19 diagnostic and prognostic classifiers, respectively. The seven fingerprint gene used are: (a) the three gene sets that characterize the progression of SCOV2 infection and resulted from the experiments with datasets GSE151513, GSE158930 and GSE148729 (tasks 1 and 3), (b) the two gene sets that characterize the low viral-load profile of infected individuals and resulted from the experiments with datasets GSE152075 and GSE156063 (task 4), and (c) the two gene sets that characterize the profile of early responders to the infection and resulted from the experiments with datasets GSE166190 and GSE161731 (task 5) (please refer to section 5). The 52 genes were assessed for their power in the diagnosis of SCOV2 infection and the prognosis of COVID-19 when used as descriptors in respective classifier models. The diagnostic classifiers aim to differentiate between SCOV2 and other (viral or non-viral) acute respiratory illness (ARIs). Three studies and respective datasets were utilized for this task, namely: GSE156063 (65), GSE188678 (72) and GSE163151 (73). The performances of the induced classifiers were compared with the results reported in the respective publications. The prognostic classifiers aim to predict the different SCOV2/COVID-19 phenotypes with respect to different clinical outcomes (e.g., severe/critical vs. mild-moderate/non-critical) or symptomatology (symptomatic vs. asymptomatic) of real patient cases. Four studies and respective datasets were utilized for this task: GSE152418 (74), GSE178967 (75), GSE172114 (76) and GSE177477 (77). The performance of the induced prognostic models was also assessed and compared with the results reported in the respective publications.

#### 2.2.2 Analysis pipeline

**Figure 2:**
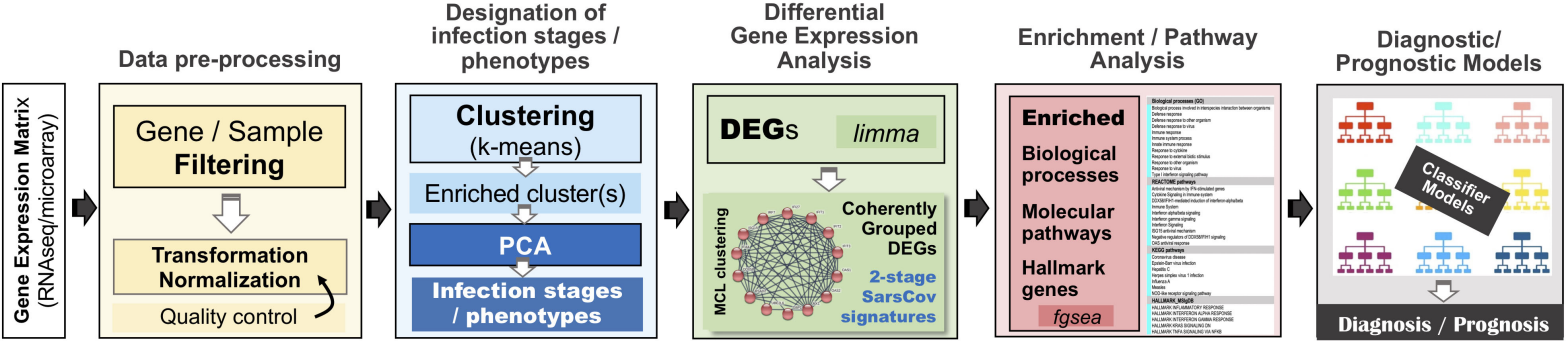
The overall analytical pipeline – operations and their flow.

For our in-silico experiments we followed a specially designed analytical workflow, the basic operational components of which are outlined in **Figure 2**. As we have already mentioned, in an effort to serve reproducible science and replication of results, at the end of the **Supplement File** (′Supplement.pdf′), a table is provided (**Supplementary Table 1**) that summarizes the specific setup for each of the performed experiment.

##### 2.2.2.1 Gene filtering and normalization

For RNAseq datasets delivered in gene count formats a minimum CPM (counts per million) cutoff was set for a predetermined number of samples (e.g., CPM ≥ 3 in half of the samples). In all other cases, a filtering of gene-probes or genes was performed according to a preset minimum expression value. For these experiments, 40% of low expressed genes (based on their maximum value across all samples) are discarded. In addition, probes or Ensembl-transcripts not mapped to HGNC official symbols are also filtered-out. Mapping of gene-probes or Ensembl transcripts to HGNC official symbols was done either by the automatic iDEP’s mapping service or, with the help of the g:profiler server^4^. Not normalized RNAseq data were normalized with DESeq’s variance stabilizing transformation/VST method (59), as implemented in the iDEP server. The specific gene filtering and normalization processes followed in each experiment were outlined in previous section 2.1.1 where, in the presentation of each task the data-preprocessing details for the respectively used datasets are presented (refer also to **Supplementary Table 1** at the end of the provided **Supplement File** (Supplement.pdf).

##### 2.2.2.2 Designation of infection stages and phenotypes

**Figure 3:**
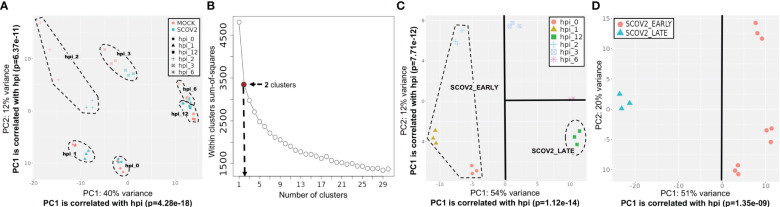
Designation of infection stages. [GSE151513]. **(A)** PCA plot of mock-treated and SCOV2 infected samples; both types of samples are grouped according to the hpi variable (the groups of samples are surrounded). **(B)** Selection of the optimal number of clusters with the Elbow method for k-means clustering. **(C)** Designation of infection stages based on the inspection of samples’ PCA plot on the cluster of 1,199 genes found enriched in immune/defense GO-biological-processes; samples (surrounded) in hpis 0, 1 and 2 designate the EARLY, and samples in hpi the 12 the LATE SCOV2 infection stage, respectively. **(D)** PCA plot of the samples (using all 9,457 genes of the filtered dataset) assigned to the respective SCOV2_EARLY and SCOV2_LATE infection stage classes; the line indicates the PC with the largest explained variance, here PC1 (51%) which is correlated with the hpi variable (p=1.35e-09).

A central component of our analysis is the identification of SCOV2 infection stages and/or phenotypes based on the available gene-expression profiles. To do so, the genes of the input dataset were clustered using the k-means algorithm. The pre-defined number of clusters was determined with the Elbow method − an effective method used by various biomedical and gene-expression analysis studies (78, 79). In all experiments, k-means was applied on a set of 500-2000 most variable mean-centered genes. The genes of each cluster were then passed through an enrichment analysis process in order to identify and focus on the cluster being heavily enriched in GO-biological-processes (geneontology.org) that relate to host immune and defense responses. With a careful inspection of the PCA plot of the samples constrained on the genes included in this cluster, the clearly separable sample groups are identified. We take these groups as representatives, either of the different infection stages (e.g., early/high) or, the different viral-load phenotype classes (e.g., high/low), with the samples of each group to be assigned to the respective infection stage or viral-load class. The specifics of the designation of infection stages or phenotypic classes followed in each experiment are detailed in the respective sections and paragraphs. For a detailed example of the methodology followed for the designation of infection stages please refer to the paragraph ′Designation of SCOV2 infection stages′ in section 3.1.1 and its supporting (**Figures 3B–D**). Such an elaborate and thorough designation of infection stages and phenotypes, represents a novel rationally designed approach for the analysis of relevant gene-expression data. Specifically, in the case of SCOV2 infection, it highlights and enables the emergence of critical differential genes and molecular fingerprints that characterize the infection.

##### 2.2.2.3 Differential expression gene analysis

For each experiment, the already normalized or VST transformed gene-expression profiles of samples are assigned to respective infection stages or phenotype classes which, were then passed to a differential expression gene (DEG) analysis process. The well-known limma R package (80, 81), as implemented in the iDEP server, was utilized for this. Limma was preferred for DEG analysis as it is applied directly on transformed/normalized gene-expression profiles and not on read counts (also suggested by the developers of iDEP). DEGs are induced by setting FDR (false discovery rate) to 0.05 and varying fold-change (FC) thresholds in the different experiments. In most cases the minimum FC threshold was set equal to 2, but for datasets with a large number of induced DEGs, and in order to ease their biological interpretation, higher FC thresholds were chosen. The specifics of DEG analysis followed in each experiment are detailed in the respective sections and paragraphs. Consult also **Supplementary Table 1** ate the end of the provided **Supplement File** (′Supplement.pdf′).

##### 2.2.2.4 Enrichment & pathways analysis

The identified up-/down-regulated DEGs were subjected to enrichment analysis for the following entries: GO-biological processes (82, 83), KEGG (www.genome.jp/kegg/pathway.html) and REACTOME (reactome.org) molecular pathways, as well as for specific gene hallmark signatures from the Human MSigDB collections (http://www.gsea-msigdb.org/gsea/msigdb/collections.jsp) (84). The Benjamini–Hochberg adjusted hypergeometric test was used and applied for the identification of enriched entries, using the fgsea R package (85) as implemented in the iDEP server.

##### 2.2.2.5 Coherent clusters of DEGs

We heavily relied on the STRING server (string-db.org, version 11.5) (86), and in particular on the respective clustering services it offers, in order to form coherent networked gene clusters from the identified DEGs. We consider and interpret these clusters of networked genes as representative core molecular fingerprints for the designated SCOV2 infection stages or phenotypes. STRING collects, scores and integrates a spectrum of publicly available sources (e.g., ENSEMBL, GeneCards, KEGG, NextProt, RefSeq and UniProt) of physical and functional protein-protein interaction information, coupling them with computational predictions, to offer operations that ease the formation of comprehensive protein/gene networks (86). Downstream analysis and interpretation of results was supported by the MCL (Markov Clustering) clustering algorithm which was applied on the STRING formed network of the identified DEGs. MCL is a fast and scalable unsupervised graph clustering algorithm (87). It has proven its superiority in extracting clusters from interaction networks (88), and its effectiveness for protein association network analysis (89). MCL is applied on the weighted network of induced DEGs, with the weight of connecting gene edges acquired from relevant information sources (text-mining, experimental, databases, co-expression, neighborhood, gene-fusion and co-occurrence). By retaining the highly confident connections (the highest cutoff of 0.9 was used) the formed robust and coherent clusters of gene networks offer informative hints about the key molecular fingerprints underlying SCOV2 infection, and serve the biological interpretation of our *findings*. Exploiting the rich sources of molecular knowledge managed by STRING to generate gene association networks, and in particular, our focus on the coherent clusters of networked genes as key molecular fingerprints underlying infection, represent a novel and at the same time, rational and well-designed analytical approach for corresponding efforts in the field. The STRING visualization services were used in order to picture the constructed gene network clusters which, were then manually adjusted to be included in the reported figures.

##### 2.2.2.6 Diagnostic and prognostic classifiers

The devise of diagnostic and prognostic classifiers is based on the union over the induced fingerprint genes that differentiate and characterize: the early SCOV2 infection stage, the low viral-load molecular profile of infected individuals, as well as the profile of early responders to the infection. This union consists of 52 unique genes that were used as descriptors for the devise of the respective SCOV2 diagnostic and COVID-19 prognostic classifier models. The Weka open-source machine-learning environment (www.cs.waikato.ac.nz/ml/weka/) was utilized for the devise and performance assessment of the classifier models (details are presented in see section 5).

## 3 Results

### 3.1 Comparison of the progression of SCOV2, SCOV1 and INFL infections: Similarities and differences

#### 3.1.1 SCOV2 follows a two-stage profile characterized by suppressed IFN-signaling and blocking of the induction of ISGs at the early stages of the infection

Initially a PCA analysis was performed on the normalized and filtered GSE151513 gene-expression data (see section 2.1.1/Tasks, datasets and set-up of experiments/′Task1′) in an effort to explore the separation between SCOV2 infected and mock-treated samples. The PCA plot showed that the two types of samples are mixed. Both mock-treated and infected samples are grouped together and according to the hpi time-points, (**Figure 3A**), with the first PC components to be strongly correlated with the hpi variable (p=4.28e-18 and p=6.37e-11 for PC1 and PC2, respectively). This indicates a strong dependence of the underlying molecular events from the progression of the infection, i.e., from early to the later post infection time-points. Focusing on just the mock-treated samples and inspecting the respective PCA plot (**Supplementary Figure 1A**), it can be observed that the samples are grouped according to the hpi variable and not according to the different replicate batches (both PC1 and PC2 components are highly correlated with the hpi variable), indicating the absence of batch effects that could be caused by replicate samples. This may be also confirmed by the respective heatmap (**Supplementary Figure 1B**) where, mock-treated samples are clearly clustered according to the hpi time-points. In addition, as we show in the sequel (at the end of the experiment with GSE151513 dataset), the molecular profile underlying the course of mock-treated samples, from the early hpi time-points to the later ones, differs drastically from the respective profile of infected samples. The profile of mock-treated samples is mainly dominated by normal cell-cycle events and not by immune/defense processes that dominate (as we showcase in the sequel) the infected samples. The non-separation of SCOV2 and mock-treated samples was further confirmed by the inability to infer any differential expressed gene to discriminate between the respective samples, even with relatively low FDR (≤0.1) and FC (≥1.5) cutoff values. The finding suggests that the molecular background underlying SCOV2 infection should be explored with respect to the progression stages of the infection. So, in the sequel we focus only on the SCOV2 infected samples and try to contrast between the different progression stages of the infection.

##### 3.1.1.1 Designation of SCOV2 infection stages [GSE151513^5^]

The designation of infection stages and the assignment of samples into respective classes was performed by following a k-means/PCA-plot process on the most variable genes (for details refer to section 2.1.2/Analysis pipeline/′Designation of infection stages and phenotypes′). (**Figure 3B**) shows the Elbow-plot where, two clusters found to be the optimal choice for k. From the two induced clusters, one (consisting of 1,199 gene-transcripts) found to be largely dominated by enriched GO-biological processes that are directly related to cell-mediated host immune/defense responses, including: ‘Response to type I interferon’, ‘Defense response to virus’, ‘Cellular response to type I interferon’, ‘Negative regulation of viral process’, ‘Response to virus’, ‘Interferon-gamma-mediated signaling pathway’, ‘Negative regulation of viral genome replication’ and ‘Regulation of viral life cycle’. (**Figure 3C**) shows the PCA plot of samples on the expression-profiles of the 1,199 cluster genes. With a careful inspection of the plot, and by taking in consideration that both PC1 and PC2 components are strongly correlated with the hpi variable (p=1.24e-14 and p=7.71e-12 for PC1 and PC2, respectively), two separable groups of samples could be clearly identified − one with samples in hpi time-points 0, 1 and 2 (n=9), and one with samples in hpi 12 (n=3). These hpis designate the respective EARLY and LATE SCOV2 infection stages, with the samples in each group to be assigned to the corresponding stage class (a total of n=12 samples). As the scope of our study is to intensely contrast between the extreme stages of the infection course, the samples at intermedia hpis 3 and 6 are discarded. The clear separation between the two infection stage classes (when the intermedia hpis are removed) could be observed by inspecting the PCA plot of the retained 12 samples on all 9,457 genes (**Figure 3D**).

##### 3.1.1.2 DEG analysis [GSE151513]

**Figure 4:**
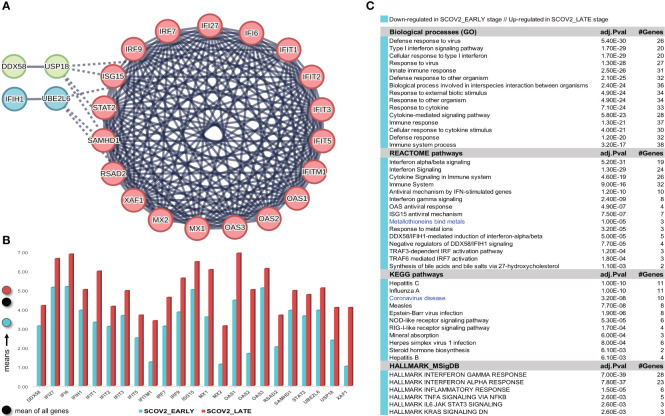
[GSE151513]. **(A)** Network of the 23 coherently clustered DEGs that compose the two-stage SCOV2 core molecular fingerprint for GSE151513 and found as down-regulated in the EARLY SCOV2 stage. **(B)** Expression levels of the 23 genes that contrast between the respective EARLY/LATE SCOV2 stages; the mean expression value of the 23 genes over the samples assigned to SCOV2_EARLY and SCOV2_LATE stages are indicated with cyan and dark-pink circles at the left of the figure, respectively; mean expression value of all genes across all samples is indicated with black-circle. **(C)** Enriched entries (GO-biological processes, REACTOME/KEGG pathways and Hallmark MSigDB signatures) found as down-regulated in the EARLY (up-regulated in the LATE) SCOV2 infection stage; cyan color indicates down-regulation; notice the down-regulation of KEGG COVID-19 pathway indicated with blue color.

The gene-expression profiles of the retained SCOV2 EARLY/LATE samples were subjected to DEG analysis. Note that the analysis is performed on all, retained after filtering, 9,457 genes, and not just on the 1,199 genes of the enriched cluster used to designate the infection stages. By setting FDR≤0.05 and FC≥2, a total of 132 gene-transcripts (DEGs) were found to significantly differentiate between the LATE and EARLY stages; 79 up- and 53 down-regulated in the EARLY infection stage. All induced DEGs induced by the experiment with the GSE151513 dataset (and for all other experiments reported in the sequel) are included in the DEGs.xlsx file that is deposited in the **Supplement Material** accompanying the paper (consult sheet ′1_GSE151513_SCOV2_2-stage′). Here it is crucial to note that the differentiation polarity is inverted, that is, down-regulated DEGs in the EARLY stage are up-regulated in the LATE stage, and vice-versa. In an effort to gain insight into the functional roles and the interactions between the 53 down-regulated DEGs we followed a clustering approach using the MCL graph-based clustering algorithm (refer to section 2.1.2/Analysis pipeline/′Designation of infection stages and phenotypes′). Keeping only high-confidence gene interactions (i.e., a cutoff of 0.9 was used in the construction of the STRING gene network), 23 DEGs, all protein coding genes, were coherently grouped into three interconnected clusters (**Figure 4A**); the three clusters are indicated with different colors). We consider these genes as representatives for the **two-stage SCOV2 core molecular fingerprint** (for the GES151513 dataset). In addition, as shown in (**Figure 4B**), these genes exhibit low and high expression levels in the EARLY and LATE infection stages, respectively; the colored-shaded circles at the left of the figure indicate the mean expression value^6^ of the 23 genes over the samples assigned to the respective infection stage (indicated with cyan and dark-pink shaded circles for the SCOV2_EARLY and SCOV2_LATE samples, respectively), including the mean gene-expression value of all genes over all samples (indicated with black shaded circle).

What is important is that all the 23 genes are directly related to IFN-I signaling and especially to ISGs. **Table 2** summarizes the functional roles and key antiviral activities of these genes. The findings agree with the discussion made in section 1/′The molecular canvas of immune and defense response during the infection course: the SCOV2 case′ about the special antiviral role of critical ISGs during the whole viral life-cycle process.

**Table 2:** Key antiviral activities of the 23 genes in the two-stage SCOV2 core molecular fingerprint being down-regulated in the EARLY SCOV2 infection stage; their functional interactions with other genes in the list as well as other engaged genes that belong to the broader families of the 23 genes are also reported (in bold) - the reported information was thoroughly and carefully collected from various established public resources.

Gene	Key antiviral activities
**IFI27/6** **IFIT1/2/3/5**	**−** Inhibit the entry of viruses to the host cell cytoplasm; prevent viral fusion and release of viral contents into the cytosol **−** Active against multiple viruses – influenza A, SCOV1&2, marburg virus/MARV, ebola/EBOV, dengue virus/DNV, west Nile virus/WNV,/HIV-1, hepatitis C virus/HCV
**IFITM1**	− Member of the IFN-inducible transmembrane (IFITM) family (**IFITM**1,**2,3,5**) − IFITM1 blocks virus entry and inhibit various viruses (e.g., Ebola, Marburg, Hepatitis C Virus/HCV) including SCOV1&2; inhibits HIV-1 production − May block membrane fusion of diverse enveloped virus
**ISG15**	− At least 158 putative ISG15 target proteins are identified, with important roles in IFN-I response − Upregulation of ISGs results in an antiviral state and reduction of viral spread − Holds a critical role of innate immune response at the initial infection stages − Acts as a cytokine to exacerbate SCOV2-triggered inflammation − Regulated by viral RNA sensors, including **IFIH1** (MDA5)
**IRF7/9**	**−** A critical regulator of IFN-Is **−** IRF9 associates with phosphorylated **STAT1**/STAT2 and promotes the transcription of ISGs **−** IFN-related STAT1 nuclear translocation proved indispensable for antiviral transduction
**STAT2**	**−** Mediates induction of IFN-Is/ISGs; associates with **IRF9** to promote the activation of ISGs **−** Mediates proinflammatory responses to **TNFα** signaling
**OAS1/2/3**	− 2’-5’-oligoadenylate synthetases (OAS) family includes OAS1, OAS2, OAS3, and OAS-like (**OASL** which is associated with viral translation) interferon-induced antiviral enzymes − Regulate the early phase of viral infection by degrading viral RNA in combination with **RNaseL**, resulting in the inhibition of viral replication
**MX1/2**	**−** Recognize the nucleoproteins or (nucleo-)capsid proteins of different viruses **−** Provide a molecular signature to distinguish between host and non-host mRNAs during viral infection **−** Sensor viral single-stranded RNAs (ssRNAs) and inhibit expression of viral mRNAs **−** MX1: potential suppressive effect on the activity of viral ribonucleoprotein complex and its GTPase **−** MX2: may be effective in repressing viral replication, transcription, and nucleocapsid shuttling
**XAF1**	**−** Stabilizes **IRF1** protein and induces more antiviral activity of IRF1 target genes, including **DDX58/RIG-I**, **DDX60**, **MX1**, and **OAS2**
**RSAD2** (Viperin)	**−** Plays major role in the cell antiviral state induced by IFN-Is; it can also be upregulated independently of IFN, through an **IRF1** or **IRF3** mechanism **−** Employs multiple mechanisms to limit the viral life cycles, especially by inhibiting viral replication at the plasma membrane **−** Has underlying anti-viral egress and replication effect
**SAMHD1**	− Restricts viral DNA synthesis, preventing virus replication by regulating innate immune sensing and mediating the (up-)regulation of IFN-Is/ISGs **−** Mediates proinflammatory responses to TNFα signaling − Negatively regulates inflammation via its interaction with various key proteins in DNA damage repair pathways
**USP18**	**−** Mediates the regulation of **ISG15** via its conjugation (ISGylation) - an enzymatic cascade of **UBE1L**/**UBAT**, **UBE2L6** and **HERC5 −** Mediates the regulation of inflammatory response to IFN-Is
**DDX58** (RIG-I)	**−** Innate immune receptor; plays a major role in sensing viral infection and in activating of a cascade of antiviral responses including the induction of IFN-Is and proinflammatory cytokines **−** Critical role in sensing CoVs (and other viruses) and the activation of ISGs **−** Regulates **IRF7/3** via the RIG-I => IRF7/3 signaling pathway
**UBE2L6**	**−** Critical role in ISG15 regulation as a modifying enzyme, in association with **USP18**, **UBAT** and **HERC5 −** Engaged in virus-induced macrophages
**IFIH1** (MDA5)	**−** Innate immune receptor; plays a major role in sensing viral infection, including SCOV2, and the induction of proinflammatory cytokines

In order to validate our findings we collected indicative sets of genes with documented antiviral activity from four relevant studies, namely: (i) a set of 42 ISGs reported in a review about the antiviral functions of ISGs (90); (ii) a set of 33 IFN-I related genes reported in a recent study from the COvid-19 Multi-omics Blood ATlas (COMBAT) consortium (www.combat.ox.ac.uk) (91); (iii) a set of 25 ISGs reported in a recent study to contrast the gene expression profiles of SCOV2 infected samples between adults and children (92); and (iv) a set of 24 key genes interfering the viral life cycle reported in (38). The union of these four gene sets comprises 97 unique genes (consult the provided **Supplement File** DEGs.xlsx/′1_GSE151513_SCOV2_2-stage′). The fact that 21 of the 23 (91.3%) genes included in the aforementioned two-stage SCOV2 core molecular fingerprint fall within this union supports our findings and provide a strong evidence for the impaired immune/defense host response taking place during the early SCOV2 infection stages.

Here we have to make a special note about three genes, MT1F, MT1G and MT2A which, although not included in the 23 fingerprint genes, they are included in the list of induced 53 induced down-regulated DEGs in the early SCOV2 infection stage (notice also the down-regulation of the enriched ′Metallothioneins bind metals′ enriched REACTOME pathway induced by the enrichment/pathway analysis presented in the sequel; (**Figure 4C**). These genes belong to the family of Metallothioneins (MTs), a family of small highly conserved cysteine-rich metal-binding proteins. MTs regulate zinc (Zn) (93) that has a beneficial role in physiological and molecular host defense mechanisms during various pathogen infections, including SCOV2 (94). *In-vitro* experiments on mice reveal a direct and strong increase in the mRNA levels of MTs during acute influenza/A infection, especially at the upper respiratory tract (95). The physiology underlying this increase is attributed to the beneficial antioxidant role of MTs as they are triggered in order to effectively ‘clean-up’ the reactive oxygen species (ROS) generated by the host defense phagocytes during infection. It is also known than Zn contributes to host defense responses by maintaining the membrane barrier structure and function (96) via the modulation of cytokine-induced epithelial cell barrier absorptiveness (97). At the molecular level, recent studies demonstrate that Zn is required for interferon-mediated expression of MTs (98), and helps to enhance IFN-I response during SCOV2 infection, exhibiting an inhibitory ability of SCOV2 RNA polymerase through its failure to be associated with serious clinical outcomes (99). Furthermore, Zn deficiencies are directly linked to anosmia and taste dysfunctions (ageusia), already established as common SCOV2 symptoms (100), especially when decreased levels occurs in the nasopharyngeal tract (101). In addition, it is evidenced that acute viral infection of the nasopharyngeal mucosa lead to a decrease in local Zn levels as part of the normal defense against respiratory pathogens (102).

##### 3.1.1.3 Enrichment analysis [GSE151513]

In order to reveal and highlight the molecular events that take place during the progress of SCOV2 infection, we proceed to the identification of enriched biological processes, pathways and hallmark gene signatures that contrast between EARLY and LATE SCOV2 infection stages. Based on the induced 132 DEGs, a number of GO-biological processes, REACTOME/KEGG pathways and HALLMARK_MSigDB gene signatures were found as significantly enriched and down-regulated in the early infection stage (**Figure 4C**). The results signify the fact that during the early stage of the infection, key biological processes and pathways engaged in first-line host innate immune/defense responses are ‘blocked’, with the list largely dominated by IFN/cytokine signaling and pro-inflammatory processes. Notably, even the KEGG COVID-19 pathway is found to be down-regulated during the early infection stage. The findings provide strong evidence that down-regulation of key antiviral immune/defense processes during the early course of SCOV2 infection and their up-regulation at the later stages may be the cause for the later emergence of uncontrolled, exaggerated and acute inflammatory COVID-19 clinical outcomes.

We performed the same as above analysis using only the mock-treated samples, with the respective samples assigned to the same infection stages, i.e., samples in hpis 0,1,2 to the early, and samples in hpi 12 to the late stage, respectively. DEG analysis resulted into 119 DEGs; 81 up- and 38 down-regulated in the early stage (data not shown). None of these genes (either up- or down-regulated) belongs to the list of the identified 23 genes that consist the formed two-stage SCOV2 core molecular fingerprint. Performing enrichment analysis of these genes, a list of fifteen significantly enriched biological processes were identified, all up-regulated in the early stage. The list was strongly dominated by cell-cycle events, including ‘Regulation of programmed cell death’, ‘Regulation of apoptotic process’, ‘Apoptotic process’, ‘Cell death’, ‘Programmed cell death’, ‘Negative regulation of cell population proliferation’, and ‘Cellular developmental process’. This highlights the fact that, even if both infected and mock-treated samples are grouped according to the hpi variable (consult **Figure 3A**), their molecular backgrounds differ drastically. In other words, the infected samples follow the cell-cycle ′norm′ but with overwhelming immune/defense molecular events being under- or over-expressed during the early or late infection stages, respectively. This proves the soundness of our approach to focus only on the SCOV2 samples and contrast between the designated early/late stages in order to explore and assess the two-stage molecular profile that governs the progression of the infection.

**Figure 5:**
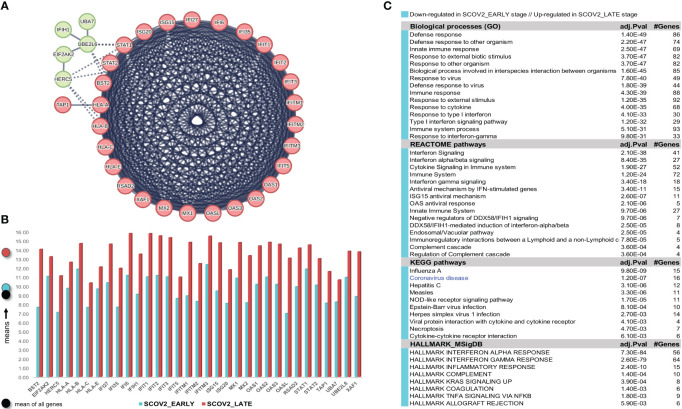
[GSE158930]. **(A)** Network of the 33 coherently clustered DEGs that compose the two-stage SCOV2 core molecular fingerprint for GSE158930 and found as down-regulated in the EARLY SCOV2 stage. **(B)** Expression levels of the 33 genes that contrast between the respective EARLY/LATE SCOV2 stages (the shaded circles at the left of the figure as in **Figure 4**). **(C)** Enriched entries (GO-biological processes, REACTOME/KEGG pathways and Hallmark MSigDB signatures) found as down-regulated in the EARLY (up-regulated in the LATE) SCOV2 infection stage; cyan color indicates down-regulation; notice the down-regulation of KEGG COVID-19 pathway indicated with blue color.

Confirmation of the two-stage SCOV2 infection profile was done using the GSE158930 RNAseq dataset (see section 2.1.1/′Task1′). Following the same methodology to identify infection stages, samples in hpi 4 and 24 (n=4) were assigned to the EARLY, and samples in hpi 72 and 96 (n=4) to the LATE SCOV2 infection stage, respectively (see **Supplementary Figures 2A–C**) for details). Setting FDR≤0.05, FC≥4 (a higher FC threshold was set in order to control the number of differential genes and ease their interpretation), and setting the ′rep′ variable as an extra factor to take in consideration the ′rep′licated/paired samples, a set of 175 DEGs (all protein coding genes) were found to differentiate between the LATE and EARLY stages (consult **Supplement File** DEGs.xlsx/′1_GSE158930_SCOV2_ 2-stage′); 15 up- and 160 down-regulated in the EARLY infection stage. Applying MCL clustering on the down-regulated genes and following the same network analysis methodology as in the previous experiment, 33 DEGs (all protein coding genes) were coherently grouped into two interconnected clusters (the two-stage SCOV2 core molecular fingerprint for GSE158930, (**Figure 5A**). Their expression levels are shown in (**Figure 5B**), and the respective enriched entries (biological processes, pathways and hallmark MSigDB signatures) in (**Figure 5C**).

The union of the two core two-stage SCOV2 molecular fingerprints identified from the previous experiments (23 genes for GSE151513 and 33 genes for GSE158930) consist of 38 genes being down-regulated in the EARLY SCOV2 infection stage, with 18 of them to be shared between the two fingerprints, namely: IFI27, IFI6 (IFI27-like/ISG12), IFIH1, IFIT1/2/3/5, IFITM1, ISG15, MX1/2, OAS1/2/3, RSAD2, STAT2, UBE2L6 and XAF1 (consult **Supplement File** DEGs.xlsx/′1_finger.Genes_UNION_COMMON′). The 18 genes and their MCL clustering are shown in **Supplementary Figure 3A**). As for the respective enriched entries (**Supplementary Figure 3B**), a set of 12 down-regulated enriched biological processes are shared between the two experiments (80.0% for both experiments); 10 REACTOME pathways (66.7% and 90.0% for GSE151513 and GSE158930, respectively); 7 KEGG pathways (63.6% and 70.0% for GSE151513 and GSE158930, respectively); and 5 hallmark MSigDB signatures (83.3% and 62.5% for GSE151513 and GSE158930, respectively). It can be easily checked that the shared enriched entries are heavily dominated by innate immune/defense processes being down-regulated during the early SCOV2 infection stage.

A specific reference should be made here concerning four genes, namely: HLA-A, HLA-B, HLA-C and HLA-E. Even if these genes are not in the list of 33 genes that compose the core SCOV2 molecular fingerprint for GSE158930, they are in the list of the induced 160 down-regulated genes in the EARLY SCOV2 infection stage. All these genes are harbored at the MHC/HLA (major histocompatibility complex/human leucocyte antigen) genome region, known as the most human polymorphic gene region. It is established that the polymorphic abundance of MCH/HLA genes is the key for their critical role in the regulation of host immune responses to most attaching pathogens (103), including SCOV2 (104). The human MHC/HLA genes are engaged to antigen processing, presentation and immune modulation. Their main functional role is to present antigens to CD8+ cytotoxic T lymphocytes (105), whereas their expression is induced by IFN genes (106). Furthermore, a number of variants in HLA alleles/haplotypes are known to be associated with susceptibility and progression of various infections, including SCOV2, with some of the most indicative summarized in (107), namely: HLA-A*11:01/24:02 with protective role against SCOV2 infection; HLA‐A*24:02 and HLA-B*22 with SCOV2 infection susceptibility; HLA-A*25:01 with moderate disease outcomes; and HLA-A*01:01/02:01, HLA-B*15:03/27:07, HLA-C*05 and HLA-E*01:01 with severe outcomes.

In their original publications the providers of GSE151513 (57) and GSE158930 (58) datasets do not directly contrast between the early and late infection stages, as we done in our analyses. In any case, in the publication of the GSE151513 dataset, the authors report a set of 19 ISGs being up-regulated in the late SCOV2 infection stage. 68.4% (13/19) of them are shared with the set of 18 genes being common between the two SCOV2 core molecular fingerprints identified in the previous experiments. Among other, two genes, STAT2 and UBE2L6, with key antiviral activities (consult **Table 2**) in the list of the 18 genes are not in the list of the 19 reported genes. As for GSE158930, in their respective original publication the authors report a set of 26 genes being up- and down-regulated in the late (72, 96 hpis) and early (24, 48 hpis) infection stages, respectively, with 42.3% (11/26) of them to be shared with the aforementioned set of 18 genes. Among other, four genes with key antiviral activities in the list of 18 genes are not included in the list of the 26 reported genes, namely: ISG15, RSAD2, STAT2 and UBE2L6. The above indicate, on one hand the competence of our analysis methodology as founded on the designation and direct contrast between early/late infection stages, and on the other, the adequacy of our findings compared to published results.

Our results provide evidence and demonstrate a two-stage core molecular profile that governs the progression of SCOV2 infection from the early to the later stages. The profile is realized by an impaired response of key immune/defense processes during the early infection stage, as materialized by the inhibition of IFN-signaling and the ′blocking′ of key ISGs, with the inverse to hold during the late stages of the infection. The findings are consistent with, and support the results reported in other relevant studies in which, the direct correlation of prolonged activation of high IFN-I levels with disease severity is highlighted (108), and the strong early IFN-I/ISG response to be beneficial for the clinical outcome of the infection (109).

#### 3.1.2 SCOV1 and SCOV2 exhibit a common core progression molecular fingerprint realized by the suppression of IFN/ISGs during the early infection stage

Both SCOV1 and MERS are known to be equipped with a variety of mechanisms to block IFN-I responses (110, 111). Several clinical studies show that both viral infections manage to escape innate immunity during the first days of infection. In particular, it is known that timing in the induction of IFNs is the key to the pathogenicity profile of SCOV1 (112, 113), with the delayed IFN-I signaling and the subsequent accumulation of monocyte–macrophages being one of the main causes for SCOV1 immunopathology (112). Furthermore, recent *in vitro* studies provide evidence that SCOV2 is sensitive to IFN-I pretreatment, even to a higher level than SCOV1 (114, 115). These findings validate and necessitate studies that contrast between SCOV1 and SCOV2 molecular profiles in an effort to explore putative common molecular fingerprints that govern both infections.

**Figure 6:**
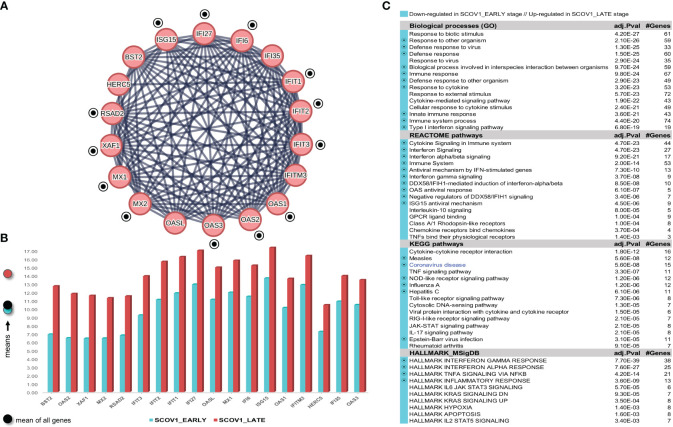
[GSE33267]. **(A)** Network of the 15 coherently clustered DEGs down-regulated in the early SCOV1 stage (the two-stage SCOV1 core molecular fingerprint for GSE33267); circled black dots indicate the 12 (from 15) genes shared with the common down-regulated genes included in the SCOV2 two-stage core molecular fingerprints resulted from the experiments with GSE151513 and GSE158930 datasets. **(B)** Expression levels of the 15 genes that contrast between the respective EARLY/LATE SCOV1 stages (the shaded circles at the left of the figure as in the previous figures). **(C)** Enriched entries found as down-regulated in the EARLY SCOV21 infection stage; cyan color indicates down-regulation; notice the down-regulation of KEGG COVID-19 pathway indicated with blue color.

As in the previous experiments, we focus only on the SCOV1 infected samples of the GSE33267 dataset (see section 2.1.1/Tasks, datasets and set-up of experiments/′Task2′) as we could not find any DEG to differentiate between mock-treated and SCOV1 infected samples. Following an analysis methodology analogous to the previous experiments, we managed to designate the early and late SCOV1 infection stages, with samples in 0, 3, 7 and 12 hpis assigned to the EARLY (n=12), and samples in 54, 60 and 72 hpis to the LATE SCOV1 infection stage (n=9). As in the previous experiments the samples at intermedia hpis, 24, 30, 36 and 48 were discarded as we are interested to study the infection progression course at its extremes. Details for the designation of SCOV1 infection stages are illustrated in (**Supplementary Figures 4A, B.1, B.2, C**). Setting FDR≤0.05 and FC≥8 (a quite high FC cutoff was set in order to keep the number of induced DEGs manageable so that we could focus on the most contrasted ones and ease their interpretation), a set of 168 DEGs to differentiate between the two infection stages were induced, 7 up- and 161 down-regulated in the EARLY SCOV1 infection stage (see **Supplementary Figure 4D**) for the respective heatmap). Applying MCL clustering on the 161 down-regulated genes and following a similar to the previous experiments’ methodology, a set of 18 down-regulated DEGs were coherently grouped into one cluster that present the two-stage core molecular fingerprint of SCOV1 infection (for GSE33267; (**Figure 6A**), see also **Supplement File** DEGs.xlsx/′2_GSE33267_ SCOV1_2-stage′). (**Figure 6B**) shows the average expression levels of these genes, with their low/high expression profiles contrasted between the respective SCOV1 EARLY/LATE stages. 13/18 genes (72.2%) are shared with the 18 genes shared between the two SCOV2 core molecular fingerprints identified in the previous experiments with GS151513 and GSE158930 datasets (down-regulated in the early infection stage); the shared genes are indicated with circled black dots. Similar findings hold for the respective enriched results (**Figure 6C**); shared enriched entries between the two infections are also indicated with circled black dots).

Confirmation of findings was done using the GSE148729 dataset (see section 2.1.1/Tasks, datasets and set-up of experiments/′Task2′). Initially we tried to contrast between SCOV1 and SCOV2 using all samples. DEG analysis, even with a low FC threshold (≥1.5), yielded no differential genes, suggesting that the contrast between the two infections should be done according to the infection progression stages. So, we followed the same as in the previous experiments methodology to designate the respective infection stages, separately for SCOV1 and SCOV2 samples. Samples in hpi 4 (n=2) and samples in hpi 24 (n=2), for both SCOV1/2 datasets, were assigned to the respective EARLY and LATE SCOV1/2 stages (see **Supplementary Figures 5A.1, A.2, B.1, B.2**)). By setting FDR≤0.05 and FC≥4, a set of 152 DEGs, 7 up- and 145 down-regulated in the EARLY SCOV1 stage, and a set of 208, 9 up- and 199 down-regulated in the EARLY SCOV2 stage were induced (see **Supplementary Figures 5C.1, C.2**) for the respective heatmaps; consult also **Supplement File** DEGs.xlsx/′2_GSE148929_SCOV1_2-stage′//′2_GSE148929_SCOV2_2-stage′). As in the previous experiments, MCL clustering was applied on the respective down-regulated DEGs; 28 for SCOV1 (the two-stage SCOV1 core molecular fingerprint for GSE148729), and 38 for SCOV2 (the two-stage SCOV2 core molecular fingerprint for GSE148729) were coherently grouped into two interconnected clusters for SCOV1, and four interconnected clusters for SCOV2, respectively (see **Supplementary Figures 6** and **7**). All 28 SCOV1 fingerprint genes are included in the respective set of 38 SCOV2 fingerprint genes. Furthermore, all genes in the union of SCOV1 fingerprint genes resulted from the experiments with GSE33267 and GSE148729 datasets (31 genes down-regulated in the early SCOV1 stage) are included in the union of fingerprint genes resulted from the experiments with GSE151513, GSE158930 and GSE148729 datasets (46 genes down-regulated in the early SCOV2 stage; consult **Supplement File** DEGs.xlsx/′2,1_finger.Genes_UNION_COMMON′). In addition, 80% (12/15) of the common fingerprint genes resulted from the experiments with GSE33267 and GSE148729 datasets (genes down-regulated in the early SCOV1 stage) are included in the list of common fingerprint genes resulted from the experiments with GSE151513, GSE158930 and GSE148729 datasets (18 genes down-regulated in the early SCOV2 stage), namely: IFI27, IFIT1/2/3, ISG15, MX1/2, OAS1/2/3, RSAD2 and XAF1. Similar results hold for the enriched entries, 83.3% biological processes, 76.5% REACTOME pathways, 62% KEGG pathways and 88.9% hallmark signatures of the union of entries from the experiments with GSE33267 and GSE148729 (down-regulated in the early SCOV1 infection stage) are included in the respective union of down-regulated in the early SCOV2 infection stage enriched entries resulted from the experiments with GSE33267, GSE158930 and GSE148729 datasets. The respective percentages for the common SCOV1 enriched entries (resulted from the experiments with GSE33267 and GSE148729 datasets) and the common SCOV2 enriched entries (resulted from the experiments with GSE151513, GSE158930 and GSE148729) are 75%, 100%, 66.7%, 66.7%. It is indicative that the respective common (and down-regulated in the respective early infection stages) hallmark entries are all related with IFN signaling, immune/antiviral responses and induction of ISGs, namely: ‘Interferon alpha/beta signaling’, ‘Interferon Signaling’, ‘Cytokine Signaling in Immune system’, ‘Immune System’, ‘Antiviral mechanism by IFN-stimulated genes’, ‘Interferon gamma signaling’, ‘OAS antiviral response’, ‘ISG15 antiviral mechanism’, ‘DDX58/IFIH1-mediated induction of interferon-alpha/beta’.

In their original publication (60) the providers of the GSE33267 dataset focus mainly on the differences between the transcriptional interferences and their effects in subsequent host immune responses caused by the wild-type SCOV1 (icSARS) or its mutant that does not express ORF6 protein (icSARS-ΔORF6). They highlight the critical role of SCOV1’s ORF6 accessory protein in antagonizing interferon signaling by blocking karyopherin-mediated nuclear import processes that enhances SCOV1 replication at the later infection stages. Even if they do not seek for differential genes and molecular processes that directly contrast between early and late SCOV1 infection stages, and so, they cannot be straightly compared with our findings, they report limited differences in differential host gene expression during the early infection stages (on 24 hpi) for both viruses. This indicate that SCOV1 (as other CoVs) invade the host cell “silently” (mainly by blocking double-stranded RNA replication intermediates and their recognition by specific pattern recognition receptors/PRRs). Regarding the original publication of the GSE148729 dataset (61), the authors report a stronger, compared to SCOV1, induction of ISGs for SCOV2 on the intermediate 12 hpi time-point, but they do not contrast between extreme infection stages (i.e., early/4 hpi vs. late/24 hpi in the setting). In that sense, the reported findings cannot be straightly compared with our findings. Even though, the authors refer and highlight the induction of IFIT1/2, OAS and IFNB1 genes (on 12 hpi), all included in the list of common genes between the two-stage SCOV2 fingerprints resulted from the experiments with GSE151513, GSE158930 and GSE148729 datasets (the 18 genes down-regulated in the early SCOV2 stage). The limited extend of the reported genes, especially IFNs, is an indication for the adequacy of our findings compared to published results.

**Figure 7:**
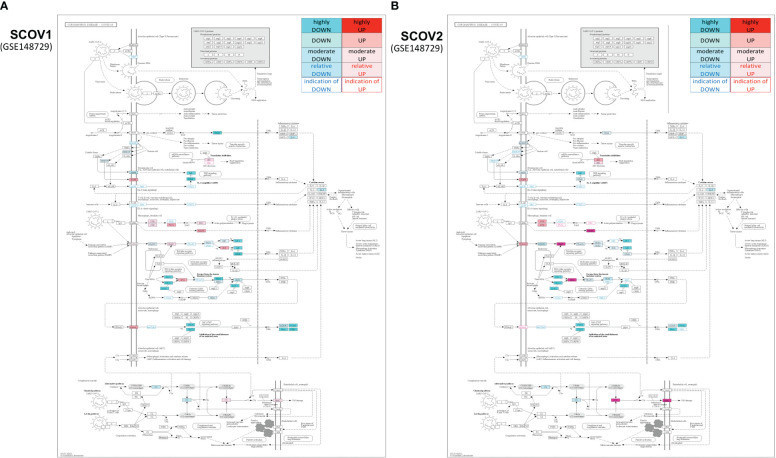
Colored KEGG COVID-19 pathways for up-/down-regulated genes in the EARLY SCOV1 **(A)** and SCOV2 **(B)** stages, both for the GSE148729 dataset − the coloring-index pallet at the top-right of the images indicate the coloring of genes according to their up-/down-regulation status and reflect (standard) deviations for the low/high values from the mean of the respective genes’ fold-changes.

Our findings demonstrate a similar core molecular fingerprint that underlies and governs the progression of both SCOV1 and SCOV2 infections, with this profile to be characterized by the blocking of core IFN-Is/ISGs during the early stages of both infections. This may be also confirmed by contrasting between the colored KEGG COVID-19 pathways of SCOV1/**Figure 7A** and SCOV2/**Figure 7B**. Coloring of genes was done with the ‘KEGG Mapper – Color’ service of the KEGG server (www.genome.jp/kegg/mapper/color.html), and it is based on the FC values of all (up- and down-regulated) induced DEGs from the respective SCOV1/2 experiments with the GSE148729 dataset. An almost identical molecular regulatory imprint between the two infections may be observed and verified.

#### 3.1.3 INFL/H1N1, compared to SCOV1/2, induce a more robust antiviral response in the early infection stages

Influenza (INFL) is the most common and long-standing viral infection worldwide with a well-established epidemiological profile and an extensive scientific literature devoted to its physiological and molecular background (116). Various studies show that symptoms from INFL infection occur already from the first day after infection, peak on the second to third day, and diminishing after five to six days (117). Severe disease outcomes are more frequent in high-risk individuals (i.e., older people with comorbidities or even younger individuals not exposed to INFL infections) and include, hospitalization, pneumonia, acute respiratory distress syndrome (ARDS), and even death. In contrast, symptoms from SCOV2 infection begin after an incubation period of about five days (at least for the strains at the early period of the COVID-19 pandemic) with the majority of cases to show symptoms for about two weeks afterward (14). According to estimates, highest transmissibility of SCOV2 occurs over a period of about four days, two days before and one day after onset of symptoms; mainly from pre-symptomatic individuals (118). A recent study documents that SCOV2 infection induces a delayed, compared to INFL-A and H1N1, transcriptional IFN responses, with slower viral replication and much lower repair responses (119). The aforementioned observations provide additional evidence to the postulate that longer incubation and manifestation periods of SCOV2, as well as longer shedding rates, result into more pre- or asymptomatic cases, making SCOV2 considerably more transmissible than INFL. The similar molecular profiles of SCOV2 and SCOV1 during the early infection stages, as showcased and highlighted in the previous section, allows us to contrast SCOV1 with INFL and draw conclusions that apply to the SCOV2 case as well, at least for the key genes and the core molecular processes underlying the two infections.

**Figure 8:**
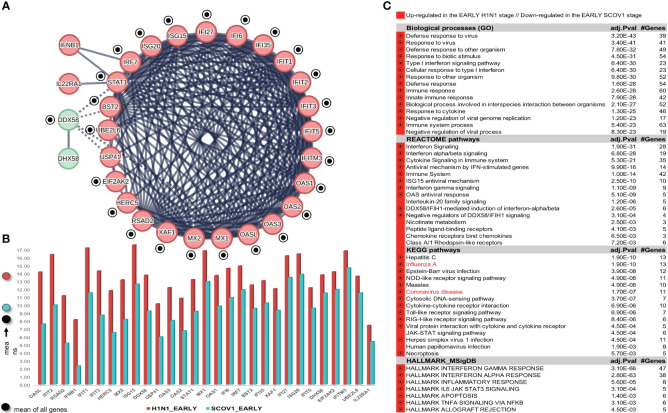
**(A)** Network of 29 up-regulated genes up-regulated (compared to their down-regulation in SCOV1/2 infections) in the EARLY H1N1 stage; circled black-dots indicate genes shared with the union of genes across the two-stage SCOV1/2 molecular fingerprints identified from all previous experiments (i.e., identified with the GSE151513, GSE158930, GSE33267 and GSE148729 dataset), and were found as down-regulated in early SCOV1/2 infection stages. **(B)** The expression levels of the 29 genes. **(C)** Enriched entries in the early H1N1 infection stage (notice the up-regulation of both Influenza/A and Coronavirus disease pathways); circled black dots indicate enriched entries shared with the union of the respective enriched entries found down-regulated in the early SCOV1/2 stage from all previous experiments (i.e., identified with the GSE151513, GSE158930, GSE33267 and GSE148729 datasets).

Designation of infection stages for the GSE47960 dataset (see section 2.1.1/Tasks, datasets and set-up of experiments/′Task3′) was done separately for H1N1 and SCOV1 samples and then, the gene-expression profiles of samples assigned to the early stages of the two infections were contrasted. Following the same methodology as in the previous experiments, H1N1 samples in 6 and 12 hpis (n=6) were assigned to the EARLY H1N1 infection stage, and SCOV1 samples in 0, 12 and 24 hpis (n=12) to the EARLY SCOV1 infection stage (see **Supplementary Figure 8** for details); all samples at intermedia hpi time-points were discarded. The designation of the early/late stages of the two infections was done by carefully inspecting the respective PCA plots (**Supplementary Figures 8B.1, B.2**), with the samples assigned to the respective EARLY H1N1 and SCOV1 stages to be separable (**Supplementary Figures 8C.1, C.2**). Setting FDR<=0.05 and FC≥4, DEG analysis contrasting between the early stages of the two infections resulted in a set of 193 DEGs, 191 up- and 2 down-regulated in the early H1N1 stage, with the reverse being true for the early SCOV1 stage (see **Supplement File** DEGs.xlsx/′3_GSE47960: H1N1_SCOV1_early′). Applying MCL clustering on the up-regulated DEGs, and after checking for duplicate probes and for gene-probes with approved HGNC gene symbols, 161 (of the 193) unique genes were subjected to MCL clustering. As a result, 29 genes were coherently grouped into two interconnected clusters [the core molecular fingerprint for H1N1 infection, (**Figure 8A**)]. (**Figure 8B**) shows the average expression level of the 29 genes in the respective early infection stages. The over-expression of these genes in the early H1N1, compared to the early SCOV1 stage, may be easily observed. Of the 29 genes being over-expressed in the early H1N1 infection stage, 25 (86.2%) also belong to the set of genes formed from the union of the respective SCOV2 fingerprint genes resulted from the experiments with GSE151513, GSE158930 and GSE148729 datasets (46 genes down-regulated in the respective early SCOV2 infection stages, consult **Supplement File** DEGs.xlsx/′2,1_finger.Genes_UNION_COMMON′); indicated with circled black dots in (**Figure 8A**). Similar results hold for the respective enriched entries (**Figure 8C**).

Here we should make a special note regarding gene IFNB1 which, even if it is in the fingerprint genes being up-regulated in the early H1N1 infection stage, it is not found to be commonly shared with the aforementioned 46 fingerprint genes being down-regulated in the early SCOV2 infection stage. It is has been shown that IFNB1 induction is significantly weaker in SCOV2 infection, with its induction in the H1N1 case occurring earlier in the infection progress and higher peaks being strongly correlated with infectious titers (119). IFNB1 is found to be associated with an increased neutrophil to lymphocyte ratio, a marker for COVID-19 late severe outcomes (120). Its production stimulates further the expression of many of the IFN-I/IFNα genes (121), enhance immune response and strongly support the resolution of viral infections and the improvement of memory responses (e.g., T and B cells), making the early up-regulation of IFNB1 beneficial for better clinical outcomes (36). Notably, IFN-Is, and particularly IFNB1, are already approved for use in the treatment of certain viral infections (hepatitis B and hepatitis C), with their administration in the early infection stages being the key for their effectiveness (120). Closely related to IFNB1 is IL22RA1, which was also found up-regulated in the early H1N1 infection stage in our experiments. Cell-line experiments have shown that IL22RA1 is induced early in H1N1 infection with its upregulation to be mediated by IFNB signaling through STAT1 (122).

In their original publication (63) the providers of GSE47960 dataset utilize both proteomic and transcriptomic datasets, coupled with other -omics resources (e.g., relevant transcription factors from the TRANSFAC database), and use the CLR relevance-based algorithm (123), to construct gene relevance networks for both SCOV1 and INFL/H1N1infections. They rank genes based on their topological positions within each of these network (e.g., centrality of a gene/node), and prioritize them based on their degree of conservation across different pathogenic infection models. Even if they do not directly compare the gene-expression profiles of samples between different infection stages, the authors report a set of 37 and 24 genes as basic regulatory features for SCOV1 and H1N1 infections, respectively, with these genes to show functional enrichment in innate immune processes. These genes share just three genes with the 29 genes in the core molecular fingerprint of H1N1 infection induced by the experiment with GSE47960 dataset, namely: IFNB1 (common with the 37 SCOV1 genes) and DDX58/RIG-I, ISG20 (common with the 24 H1N1 genes). So, compared to our analysis methodology and findings, the aforementioned gene relevance network methodology fails to identify not only an extended spectrum of key IFN/ISGs that differentiate between H1N1/SCOV1 early stages, but also, their up/down regulation status in the course of the infection progress. Such a fail indicates the adequacy of our finding compared to the published results.

Our findings demonstrate that, in contrast to SCOV1/2 infections, H1N1 drives quite early to an antiviral-state, guided mainly by early IFN-signaling and induction of antiviral ISGs. This may be attributed to the fact that INFL/H1N1 infection results into rapid viral replication leading to significant epithelial damage and to significantly greater immune responses. In contrast, SCOV2 is characterized by slower replication rates, a fact that explains the longer incubation periods of SCOV2 infection, and so, limited changes in the morphology and composition of the epithelial structure. As a result, repair responses are much weaker in SCOV2 compared to H1N1 infected cells (119). So, the question is if and how viral-load levels engage with the standard (as demonstrated by the previous experiments) two-stage molecular profile of SCOV2 infection.

## 4 How viral-load and early host response relate to the two-stage SCOV2 infection profile?

### 4.1 Low viral-loads associate with suppressed immune response in the early SCOV2 infection stage

The ways that SCOV2 acquires high viral-loads, even without the presence (or too mild) symptoms, still remain unclear. One study, already from the early period of the onset of COVID-19, suggested that the diagnostic value of SCOV2 viral-load is higher in non-hospitalized patients and inversely correlated with both the duration of symptoms and the severity of the disease (124). A recent report from the IMPACC prospective longitudinal study documented that SCOV2 viral-load and its persistence are clearly associated with severe disease outcomes (125). Lower SCOV2 viral-loads have also been shown to be associated with longer disease duration (118). Various relevant explanations and physiological models are reported for this, including the synergy between the initial number of foci-of-infection (FOI) and the varying dynamics of CD8^+^ T-cell responses (126) that drive lymphocytopenia and lead to severe and critical clinical outcomes (127). It is established that for SCOV2, like other airborne infections, the nasal microenvironment plays a central role in the early infection phases and guides the modulation of subsequent immune/defense responses (128), with a recent study showing that upper respiratory symptoms (sore throat, nasal discharge, dysosmia, dysgeusia) are good prognostic factors, in contrast to lower respiratory symptoms (cough, sputum production, dyspnea) (129). Relevant studies demonstrate the presence of high viral-loads at the nasal epithelium and mucosa during the early infection stage and after the onset of symptoms, with higher loads detected in the nose than in the oral cavity (130). Based on the aforementioned observations, the following experiments aim to contrast between the molecular fingerprints underlying different SCOV2 viral-load levels, and associate them with the two-stage progression profile of the infection.

**Figure 9:**
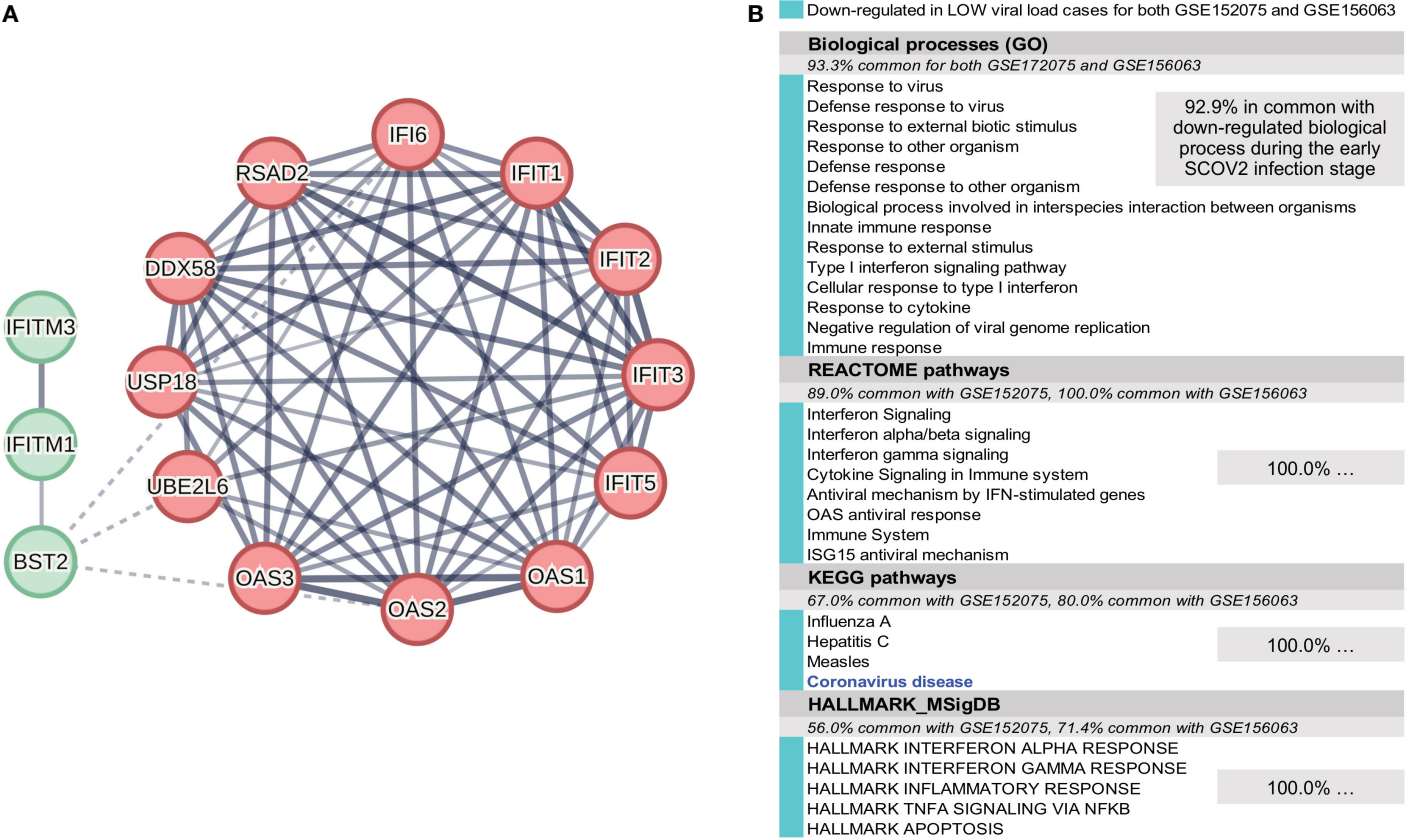
**(A)** The 18 down-regulated genes in the low viral-load cases shared with the down-regulated genes in the early SCOV2 infection stage resulted from the union of the respective two-stage core molecular fingerprints resulted from the experiments with GSE151513 and GSE158930 datasets. **(B)** Enriched down-regulated biological processes, pathways and hallmark genes common between the low viral-load cases and the union of the respective enriched down-regulated entries in the early SCOV2 infection stage resulted from the experiments with GSE151513 and GSE158930 – the percentages of the respective common entries are also indicated.

Following a DEG analysis on the GSE152075 dataset (see section 2.1.1/Tasks, datasets and set-up of experiments/′Task4′), and setting FDR≤0.05 and FC≥2, a set of 301 DEGs were induced, of which 18 were up- and 283 down-regulated for the LOW viral-load class (consult **Supplement File** DEGs.xlsx/′4_GSE152075_SCOV2_ViralLoad′). Applying MCL clustering on the network of the 283 down-regulated DEGs, a set of 26 genes were coherently grouped into three interconnected clusters [the SCOV2 core molecular fingerprint for the low viral-load level for GSE152075, (**Supplementary Figure 9A**)], with the expression levels of these genes to significantly differentiate between the corresponding viral-load levels (**Supplementary Figure 9B**). In addition, a series of biological processes, pathways and hallmark genes were found to be significantly enriched to IFN-signaling and innate immune/defense responses (**Supplementary Figure 9C**). To further confirm our findings, we performed DEG analysis on the GSE156063 dataset (see section 2.1.1/Tasks, datasets and set-up of experiments/′Task4′). Setting FDR≤0.05 and FC≥4, a set of 56 DEGs were found to be down-regulated for the LOW viral-load class (no up-regulated genes were induced), with 19 of these genes to be coherently grouped into three interconnected clusters after applying MCL clustering [the SCOV2 core molecular fingerprint for the low viral-load level for GSE156063, (**Supplementary Figure 10A**)], with the expression levels of these genes to differentiate between the corresponding viral-load levels (**Supplementary Figure 10B**). Enrichment results are shown in (**Supplementary Figure 10C**). The two core molecular fingerprints for the low viral-load level resulted from the previous experiments share 15 down-regulated genes in common; their MCL coherent clustering is shown in (**Figure 9A**). All of them (100%) belong to the set of 46 genes formed from the union of SCOV2 fingerprint genes down-regulated in the early stage. resulted from experiments with GSE151513, GSE158930, and GSE148729 datasets. Similar results hold for the respective enriched entries (**Figure 9B**).

In their original publication (64) the providers of GSE152075 dataset report a set of 363 DEGs that differentiate between high and low viral-load levels, with the 100 most differentiable (FC≥2) to be down-regulated for the low viral-load cases. MCL clustering of the 100 genes resulted into 23 genes grouped into two coherent clusters (data not shown), with 10 of them (43.5%) to be shared with the 26 genes that compose the SCOV2 core molecular fingerprint of the low viral-load level for GSE152075. But, in the remaining 16 fingerprint genes there are genes with critical antiviral roles, e.g., IFITM1 and ISG15 (refer to **Table 2** for their antiviral activities). Regarding GSE156063, the providers of the dataset report a set of 121 genes that differentiate between ARIs caused by SCOV2, other or no-virus infections, with 116 of them to exhibit an FC≥2 (across the different pairwise comparisons). They also report the slopes of the regression fitting-lines for the scatter plots of normalized gene counts as a function of SCOV2 viral-load, with higher slope values to indicate the down-regulation status of the respective genes for the low viral-load cases. In addition, they report a slope of 0.58 for gene IRF7 which, among other leading-edge interferon response genes, lag expression in SCOV2 infection compared with other ARIs. A set of 25 genes are in common between the aforementioned 116 genes and the ones with slopes over 0.58. The 25 genes share just 9 (36%) with the 19 genes that compose the SCOV2 core molecular fingerprint for the low viral-load cased induced from the experiment with GSE156063 dataset. Again (as in the case of GSE152075), in the remaining 10 genes of the fingerprint there are genes with critical antiviral roles, e.g., IFITM1, MX2 and UBE2L6. The above indicate again the adequacy of our findings compared to published results.

Our findings showcase that the molecular profile of SCOV2 low viral-load level is related to the molecular profile that characterize the early stage of the infection. As the acquisition of viral-loads, for both of the above experiments, was done at the early stages of the infection, the findings indicate an association between low viral-loads and suppressed immune responses during the early SCOV2 infection stage. This is in accordance with results presented in other relevant studies. In particular, in a study that links SCOV2 viral-load at the upper respiratory tract with disease clinical outcome it was found that patients with mild symptoms showed significantly highest viral-loads compared to severe patient cases that showed the least (131). However, it is shown that disease severity is not associated with viral-load (132), but rather to IFN responses being significantly lower in severe cases during the early infection stage compared to the mild/moderate ones (132). This drives us to the question if early immune/defense responses to the infection, as may be indicated by the early onset of symptoms, is linked with viral-loads, and how it relates to the two-stage SCOV2 infection profile.

### 4.2 Early responders exhibit a robust antiviral immune response in the early stages of infection which inverses the standard two-stage SCOV2 infection profile

It has been established that different individuals may exhibit different response profiles during the SCOV2 infection course. This is attributed to different factors including viral-load. It is shown that high viral-loads induce robust IFN responses at the early stage of the infection, with protective results against severe outcomes (131). Furthermore, many studies demonstrate that cross-immunity from the infection with other CoVs is beneficial for the disease outcome (133–136). In addition, it has been also shown that COVID-19 patients with mild/moderate disease exhibit IFN-I responses at the early infection stages (39, 137).

To explore the response profiles of individuals we used the GSE166190 dataset (see section 2.1.1/Tasks, datasets and set-up of experiments/′Task5′). Designation of early response class: In the original publication of the study for GSE166190 dataset (70), the authors report that direct gene-expression comparisons between children and adults show stronger and persistent innate inflammatory responses in adults in the first two weeks (0-5 and 6-14 DPOS), with specific B-cell responses to be significantly overexpressed in children. As a final conclusion the authors state that children and adults follow similar innate responses to the infection but with a faster resolution in children, as indicated by B-cell responses during the later infection stages. The up-regulation of biological processes related to B-cell responses is also confirmed by our analysis when directly contrasting the RNAseq profiles of the two age groups (data not shown). But the stronger and persistent IFN responses is not so profound. The comparison of the different DPOS intervals (1, 2, 3 and 4) with the last interval of the infection course (i.e., 36-81 post infection days), as performed in the aforementioned study, is not adequate for the exposition of the key molecular mechanisms underlying the infection during its active state; as it is natural to assume that 36 DPOS the infection is already in retreat. In the light of the aforementioned observations we followed a cautious process in order to identify groups of infected individuals that exhibit different immune/defense immune profiles during the early infection stage, regardless of their age. To this end, we focus on individuals (both adults and children) with early onset of symptoms (i.e., with 0-5 DPOS, n=16, 9 adults and 7 children). Applying k-means clustering on these samples (see **Supplementary Figure 11** for details) we were able to identify one cluster (consisting of 178 genes) being heavily enriched in immune/defense biological processes (see **Supplementary Figure 11B**) for the heatmap of these genes over the 16 samples). By manually inspecting the heatmap, the samples with contrasted expression profiles were assigned to two groups: earlyDOWN (n=9; 5 children/CHILD_1,2,4,5,7 and 4 adults/ADULT_1,2,4,6) and earlyUP, i.e., the early responders (n=7; 2 children/CHILD_3,6 and 5 adults/ADULT_3,5,7,8,9) with down/up-regulated gene expression profiles, respectively (in **Supplementary Figure 11B**) the green/dark-green and red/dark-red patterns designate the respective earlyDOWN/earlyUP groups; the enriched GO biological processes of this cluster are also shown). The clear separation of the two groups is confirmed by the PCA plot of the respective samples (**Supplementary Figure 11C**); the ages of the individuals assigned to the two groups are also shown). DEG/Enrichment analysis: Applying DEG analysis to contrast between the two groups, and setting FDR≤0.05, FC≥2, a set of 161 DEGs, all up-regulated in the earlyUP group, were induced (with no down-regulated genes being induced). See **Supplementary Figure 11D**) for the respective heatmap, and consult also **Supplement File** DEGs.xlsx/′5_GSE166190_ SCOV2_EarlyResponse′). MCL clustering of the 161 genes resulted into a complex of 28 genes grouped in to three interconnected coherent clusters [the SCOV2 early responders’ core molecular fingerprint for GSE166190, **Supplementary Figure 12A**)]. The contrasted expression levels of the 161 genes are shown in (**Supplementary Figure 12B**), and the respective enriched entries in (**Supplementary Figure 12C**). The findings demonstrate that there are SCOV2 infected individuals which, regardless of their age, exhibit robust immune/defense responses during the early infection stages.

To confirm our findings, we used the GSE161731 dataset (see section 2.1.1/Tasks, datasets and set-up of experiments/′Task5′). As in the previous experiments, we applied k-means clustering in order to designate groups of samples that contrast between the onset_hosp classes (see **Supplementary Figure 13** for details). Designation of early-mid response class: With a careful inspection of the respective samples’ PCA plot, two separable groups of samples could be identified (**Supplementary Figure 13B**), one with EARLY_YES and MID_YES samples (denote as ′EarlyMid_YES′, n=11 samples), and one with MID_NO and LATE_NO samples (denoted as ′MidLate_NO′, n=25 samples). The EARLY_NO cases were discarded as they are mixed between the two groups. (**Supplementary Figure 13C**) shows the PCA plot of the samples when assigned to the EarlyMid_YES and MidLate_NO groups; PC1 indicates a significant separation between the two groups (p=1.40e-6). Here we have to make an important note that relates to the infection’s clinical outcome. The hospitalization of patients in the EarlyMid_YES group does not necessarily mean that these patients are in risk for severe disease outcomes. We postulate that it is mainly their early response to the infection and their putative elevated symptomatology that leads to this medical decision. Under this interpretation, the key question concerns the underlying molecular background that guides and governs patients with early response to the infection, in contrast to patients which, even if they were not hospitalized, exhibit a late response to the infection, with putative milder symptoms at the early stage. DEG/Enrichment analysis: DEG analysis was performed by contrasting between the EarlyMid_YES and MidLate_NO groups. Setting FDR≤0.05 and FC≥2, a set of 311 DEGs were induced, 250 up- and 61 down-regulated in the EarlyMid_YES cases. Applying MCL clustering on the 250 up-regulated genes, a set of 22 genes were coherently clustered into two interconnected groups (the SCOV2 early-mid responders’ core molecular fingerprint for GSE161731; **Supplementary Figure 14A**), consult also **Supplement File** DEGs.xlsx/′5_GSE161731_SCOV2_EarlyResponse′). The 22 genes exhibit higher expression levels in EarlyMid_YES cases compared to the MidLate_NO ones (**Supplementary Figure 14B**). The respective enriched up-regulated entries are shown in (**Supplementary Figure 14C**). The 22 fingerprint genes share 86.4% (19/22) genes with the 28 fingerprint genes for the earlyUP group induced from the previous experiment with GSE166190 dataset (indicated with circled black-dots in (**Supplementary Figure 14A**); consult also **Supplement File** DEGs.xlsx/′5:UNION_COMMON′), namely: BST2, EIF2AK2, HERC5, IFI6/35, IFIT1/2/3/5, IFITM1, IRF7, ISG15, MX1, OAS1/2/3/L, RSAD2 and XAF1, with all of them to fall in the union of SCOV2 fingerprint genes resulted from the experiments with GSE151513, GSE158930 and GSE148729 datasets (46 genes down-regulated in the early SCOV2 stage), and 84.2% (16/19) of them to be shared with the union of fingerprint genes resulted from the experiments with GSE152075 and GSE156063 datasets (30 genes down-regulated for the LOW viral-load cases), namely: BST2, EIF2AK2, HERC5, IFI6, IFIT1/2/3/5, IFITM1, ISG15, MX1, OAS1/2/3/L, RSAD2.

In their original publication (70) the providers of GSE166190 dataset report enriched blood transcriptional modules (BTMs) that differentiate between the response profiles of children and adults in the early infection stage (0-5 DPOS). In particular, they report three such BMTs that include genes being related to IFN antiviral responses and which, are up-regulated in adults, namely: antiviral IFN signatures (BTM: M75), IFN-I response (M127) and viral sensing & immunity; IRF2 targets network (II) (M11.1)^7^. A set 35 genes compose the union of genes included in these BTMs with, 31.4% (11/35) to be shared with the 28 genes that compose the SCOV2 core molecular fingerprint for early responders (up-regulated for the earlyUP group) identified from the experiment with GSE166190 dataset. From the list are missing genes with critical antiviral activity, as for example: IFI6, ISG15, MX1/2, UBE2L6 and XAF1. As for GSE161731, in their original publication (71) the providers of the dataset present a set of 28 ISGs that, even if their expression profiles in the early infection stages display a similarity to those of other viral ARIs, they are muted is the case of SCOV2 infection compared to other seasonal CoVs. These genes share 15 (53.6%) in common with the 22 fingerprint genes found in the experiment with GSE161731 dataset. Again, from the list are missing genes with critical antiviral activity, e.g., IFIH1, IFITM1/3, IRF7, ISG20, MX2, STAT1/2, UBE2L6 and USP18, which are included in the list of 22 fingerprint genes for early-mid responders resulted from the experiments with GSE161731 dataset. Again, the above indicate the adequacy of our findings compared to published results.

Our findings demonstrate that early responders: (i) are able to inverse the standard two-stage SCOV2 infection profile, with key antiviral genes and molecular processes to be up-regulated during the early infection stage, and (ii) their molecular immune/defense profile resembles the respective profile of high viral-load cases. As the published data of the two studies with GSE166190 (70) and GSE161731 (71) do not refer to the viral-load of the infected individuals, our last statement should be kept as an evidenced hint. Even if pre-existing cross immunity by other HCoVs (human coronaviruses) to SCOV2 infection is debatable, there is accumulated evidence that HCoV-specific SCOV2 cross-reactive CD4^+^ T cells may trigger and enhance humoral immune responses in SCOV2 infection (138). As more than 90% of the population is HCoV sero-positive, the above may provide an explanation for the early immune antiviral responses displayed by early responders to the infection. We have already mentioned that early response to the infection could be proved beneficial for the retreat of the infection, as the controlled regulation of ISGs in the later stages allows host tolerance processes to take over. In other words, early responders, even with elevated symptoms, may have a good prognosis regarding the clinical outcome of the infection.

## 5 SCOV2 diagnostic and prognostic modelling: a machine learning approach

Risk assessment for COVID-19 infected individuals about severe/critical clinical outcomes is crucial not only for the early identification of patients that require urgent clinical care but also, for the determination of the most appropriate and effective treatment to be followed in order to avoid fatal outcomes. In addition, risk-stratification decision aids may provide valuable support for the most appropriate allocation of the needed specialized clinical wards (e.g., emergency units and ICUs). So, the devise and assessment of reliable and robust COVID-19 prognostic models raise as a major need, especially in periods of high infection rates where the national and regional healthcare systems reach their limits. Most of the COVID-19 prognostic models reported in the literature, especially for hospitalized patients, base their predictions on demographics (with age and sex as the most determinant variables), comorbidities (with special focus on hypertension, cardiovascular disease, hypertension and diabetes), laboratory indicators (e.g., lymphocyte/platelet counts, creatinine, interleukin 6 (IL-6), procalcitonin (PCT), d-dimer, ferritin etc.) (139, 140) and medical imaging (141). In a recent systematic and extensive review about the various COVID-19 prognostic models presented in the literature, the divergence between the reported performance statistics is highlighted (142). Actually, in 60 studies that refer to the severity or criticality of the disease, AUC (an indicator of the prediction robustness) ranges from 0.57 to 0.99, with sensitivity and specificity to range between 7.1% -100% and 19.5% -100%, respectively. The large deviations between predictions should be attributed to the heterogeneous criteria for disease severity/criticality followed by the different studies, a fact that puts a strong bias on the selection and stratification of patients. In addition to the above approaches, a series of studies aim to tackle the task of COVID-19 prognosis on the basis of patients’ transcriptomic/gene-expression profiles, both at the early pandemic period (78) and at the later periods (76, 143–145). In terms of COVID-19 diagnosis and gene-expression profiling the relevant efforts focus mainly on the differentiation between acute respiratory illness/ARI caused by SCOV2 or other viral/non-viral infections (65, 72, 73).

### 5.1 Fingerprint genes as classifier descriptors

**Figure 10:**
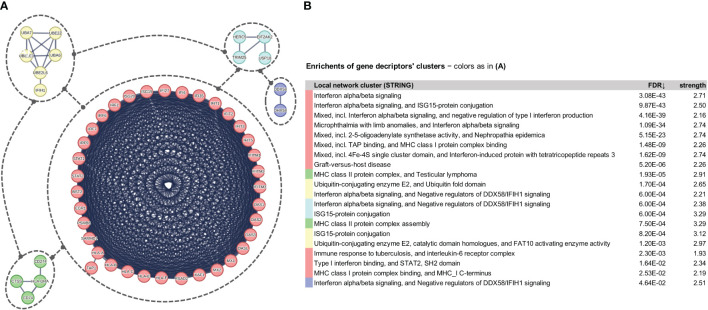
**(A)** Coherent clusters of the 52 unique fingerprint genes used as descriptors for the devise of diagnostic and prognostic classifiers. **(B)** Enrichments of the 52 genes according to the STRING/local network clusters annotations.

In the previous sections we identified a set of key-genes, dominated by IFN related genes and ISGs as the core molecular fingerprints underlying, either the early/late SCOV2 infection stages or, the different SCOV2 infection phenotypes (low/high viral-load, early/late responders). Taking advantage of these findings, we followed an intuitive approach for the devise of the classifiers’ predictor set of genes with the aim to serve and support diagnostic and prognostic decision making. The set is formed solely by the core molecular fingerprint genes induced from the tasks being directly correlated, on one hand, from the infection’s progression molecular profile, and on the other, from the host’s viral-load and response molecular profiles. So, seven fingerprint gene sets were utilized from the following tasks and respective experiments: (a) the two-stage SCOV2 progression profile, i.e., 23, 33 and 38 fingerprint genes (down-regulated in the early SCOV2 infection stage) from the experiments with GSE151513, GSE158930 and GSE148729 datasets, respectively (sections 3.1.1, 3.1.2); (b) the low SCOV2 viral-load molecular profile, i.e., 26 and 19 fingerprint genes (down-regulated for the low viral-load class) from the experiments with GSE152075 and GSE156063 datasets, respectively (section 4.1); and (c) the molecular profile of SCOV2 early responders, i.e., 28 and 23 fingerprint genes (up-regulated for the early responders to the infection) from the experiments with GSE166190 and GSE161731 datasets, respectively (section 4.2). The union over the seven gene sets consists of 52 unique genes which are used as predictors for the devise of both diagnostic and prognostic classifiers (consult **Supplement File** DEGS.xlsx/′6_CLASSIFIER_Descriptors_Enrich′ for all the details). MCL clustering of the 52 descriptor genes is shown in (**Figure 10A**); five interconnected coherent clusters. Their enrichments, provided by STRING’s local network clusters annotations, are shown in (**Figure 10B**), and indicate that the utilized descriptors are "well informed" about the core molecular events taking place during SCOV2 infection. A special note should be made concerning gene IFI27. IFI27 is found as differentially expressed in most of the core molecular fingerprints induced by the conducted experiments in the previous sections, and so, its role is central not only to the pathogenesis but also, for the diagnosis and prognosis of the disease. Further evidence for the crucial role of IFI27 is provided in a large-scale nested case-control diagnostic accuracy study where, IFI27 is suggested as a marker for the early detection of SCOV2 infection and for "abortive infection" events (146).

For the devise of both the diagnostic and prognostic classifier models we used the Weka open-source machine-learning environment (www.cs.waikato.ac.nz/ml/weka). Extensive experimentation showed that Random Forests (RF) classifiers exhibit the best performance and so, the reported performance figures for our proposed classifiers refer to the respective RF-based models.

### 5.2 Diagnostic classifiers

The diagnostic task refers to the gene-expression based differentiation of acute respiratory illness (ARI) caused either by SCOV2 (POS class) or other viral/non-viral infection (NEG class). In three studies, the results of which are reported in (65, 72, 73), different classifiers are devised to differentiate between ARIs caused by SCOV2 (POS) or other viral/non-viral (NEG) cases. We built an RF-based classifier utilizing the aforementioned 52 signature genes for each of the three datasets used in the aforementioned studies (GSE156063, GSE188678 and GSE163151, see **Table 3**), and contrast their performance with the corresponding performance figures reported in the respective publications. SMOTE (Synthetic Minority Oversampling Technique) (147) was applied on each dataset in order to overcome the class imbalance between POS and NEG samples. The gene-expression values were discretized separately for each dataset in three intervals that reflect the low, medium and high expression state of each gene; an equal width binning discretization process was followed^8^. Each classifier was assessed according to the different cross-validation procedures followed in the respective studies (i.e., 5-fold, 70% vs. 30% or 80% vs. 20% random splitting), and taking the average over 100 iterations of 5-fold or random-splitting runs. The results are summarized in **Table 3**, and showcase that the proposed classifiers outperform in most of the cases the figures reported in the respective studies, achieving robust performance, mainly indicated by the AUC figures across the majority of performance metrics; relative lower performance for specificity/SP (in some cases) and NPV may be attributed to the application of the SMOTE class balancing process.

**Table 3:** Diagnostic classifiers.

Dataset/ref/#Genes/Classifier	AUC	ACC	SE	SP	PPV	NPV
GSE156063/(65)/27/RF**¹**	0.981	92.3%	**95.7%**	**95.0%**	92.1%	**96.9%**
Proposed**^2^**	**0.984**	**94.1%**	93.6%	94.5%	**94.5%**	93.7%
GSE188678/(72)/10/SVM**^3^**	0.934	nr*	88.9%	92.8%	81.5%	**95.1%**
Proposed**^4^**	**0.982**	93.5%	**93.3%**	**93.7%**	**93.8%**	93.4%
GSE163151/(73)/1014,66,19/SVM**^5^**	0.933	86.5%	78.6%	**93.5%**	nr	
Proposed**^6^**	**0.980**	**90.9%**	**94.9%**	86.8%	87.9%	94.6%
Average (study datasets)	0.949	89.4%	87.7%	93.8%	86.8%	**96.0%**
Average (proposed)	**0.982**	**92.8%**	**93.9%**	**91.7%**	**92.1%**	93.9%

**¹**RF, Random Forest; A 5-fold cross validation performance assessment is followed in the original publication [consult (65)]; the reported figures refer to the 27-genes classifier models with the following cutoffs thresholds used: 0.4 or 0.5 for ACC, 0.4 for SE and NPV, 0.5 for SP, PPV. **^2^** The proposed method is also based on RF; SMOTE was applied to take-care class imbalances; the reported figures are the averages over 100 iterations of 5-fold runs; the reported performance figures refer to the POS (i.e., SCOV2 infection) class.

**^3^** SVM, support vector machines; A 70%/30% train/test splitting for performance assessment is followed in the original publication (consult [72)]; the reported figures refer to 10 genes resulted from the union of all 2-gene classifier models reported in the original publication; the performance figures for AUC and SE corresponds to the {IFI6, RGINA} 2-gene model, for SP and PPV to {IFI6, GBP2} and for NPV to the {IFI6, C15orf48}. **^4^** The proposed method is also based on RF; SMOTE was applied to take-care class imbalances; the reported figures are the averages over 100 iterations of 70%/30% random splits; the reported performance figures refer to the POS (i.e., SCOV2 infection) class.

**^5^** A 80%/20% train/test splitting for performance assessment is followed in the original publication [consult (73)]; the reported figures refer to the combined classifier [layer-I (POS/SCOV2 vs. NEG/non-viral ARIs) followed by layer-II (POS/SCOV2 vs. NEG/viral ARIs)] with, ACC and AUC to correspond to the full gene panel (1014 genes), SP to the full or the medium gene panel (66 genes), and SE to the small gene panel (19 genes). **^6^** The proposed method is also based on RF; SMOTE was applied to take-care class imbalances; the reported figures are the averages over 100 iterations of 80%/20% random splits; the reported performance figures refer to the POS (i.e., SCOV2 infection) class.

*nr, not reported.

Performance of diagnostic classifiers built with the set of 52 fingerprint genes and their comparison with published results (refer to section 5/′Fingerprint genes as classifier descriptors′). The devise and performance assessment of the proposed classifiers follows the same methodology followed by the respective comparison publications, i.e. 5-fold (for GSE156063), 70% vs. 30% (for GSE188678) and 80% vs. 20% random splitting (for GSE163151); the reported figures for the proposed classifiers are the averages over 100 iterations of each 5-fold run or random split. SMOTE 2760 was applied on all datasets in order to take-care for class imbalances. Bold figures indicate superior performance. AUC: Area Under the Curve; ACC: Accuracy; SE: Sensitivity; SP: Specificity; PPV: Positive Predictive Value; NPV: Negative Predictive Value.

### 5.3 Prognostic classifiers

We explored the power of prognostic classifiers to differentiate between the diverse of SCOV2 clinical outcomes (e.g., severe/critical vs. mild-moderate/non-critical) or phenotypes (e.g., symptomatic vs. asymptomatic). Utilizing the aforementioned 52 signature genes, we built classifiers for the datasets and the respective differentiations undertaken in four indicative studies: GSE152418 (74), GSE178967 (75), GSE172114 (76) and GSE177477 (77) (see **Table 4**). SMOTE was applied on each dataset, and the gene-expression values were also discretized, separately for each dataset. The performance of the classifiers was assessed via 5-fold cross-validation, taking the average over 100 iterations of 5-fold runs. The performance results are presented **Table 4**. The proposed classifiers achieve a very good and robust performance for all performance metrics across all datasets and respective differentiation tasks.

**Table 4:** Prognostic classifiers.

Dataset/*Phenotype-*1 vs. *Phenotype-2*	AUC	ACC	SE	SP	PPV	NPV
GSE152418/*SevereICU* vs. *Moderate*	0.992	95.4%	96.8%	94.1%	95.7%	97.6%
GSE178967/*Severe* vs. *Moderate*	0.997	98.9%	99.1%	98.7%	98.8%	99.1%
GSE172114/*Critical* vs. *NonCritical*	0.940	90.3%	91.2%	89.4%	90.2%	91.9%
GSE177477/*Symptomatic* vs. *Asymptomatic*	1.000	95.7%	91.6%	100.0%	100.0%	93.3%
Average (study datasets)	0.982	95.1%	94.7%	95.6%	96.2%	95.5%

Performance of prognostic classifiers built with the set of 52 fingerprint genes (see section 5/`Fingerprint genes as classifier descriptors`). Performance assessment of the proposed SCOV2 RF-based prognostic classifiers follows a 5-fold cross-validation assessment; the respective performance figures are the averages over 100 iterations of each 5-fold run and correspond to the severe class, i.e., *SevereICU*, *Severe*, *Critical* and *Symptomatic* for GSE152418, GSE178967, GSE172114 and GSE177477 datasets, respectively. Bold figures indicate superior performance. SMOTE was applied on all datasets in order to take-care for class imbalances. AUC, ACC, SE, SP, PPV and NPV as in **Table 3**.

The reported results demonstrate the feasibility of applying gene-expression profiling for the induction of reliable and robust SCOV2 diagnostic and prognostic models, especially when the utilized gene signature descriptors are well-informed about the core molecular fingerprints underlying SCOV2 infection.

## 6 Discussion

In this paper, we performed differential expression and enrichment/pathway analysis utilizing a diverse of public-domain gene-expression datasets from respective well-documented studies associated with SCOV2 infection. We posted some challenging biological questions to uncover the molecular landscape underlying and governing the infection. We attempt to provide answers to these questions and tackle the respective tasks following a multi-step Bioinformatics pipeline realized by the utilization of state-of-the-art gene-expression and pathway analysis methodologies, services and tools. To this end, our in-silico quest provide the following contributions: (i) an elaborate analytical methodology to segregate SCOV2 early and late infection stages, providing a set of, down-regulated in the early stages of the infection, key IFN/ISG genes and respective biological processes, pathways and hallmarks signatures that present the core molecular fingerprint of the two-stage SCOV2 infection profile; (ii) provide strong evidence and support the hypothesis that SCOV1 follows a similar to SCOV2 infection progression profile; (iii) contrast and differentiate between INFL/H1N1 and SCOV1/2 infections at their early progression stages and showcase a more robust, compared to SCOV1/2, antiviral host response for the case of H1N1 during the early infection stage; (iv) link low viral-load levels with the two-stage SCOV2 progression profile, providing evidence that low viral-loads at the early infection stage are associated with suppression of key IFN/ISGs; (v) designate and characterize the molecular profile of different SCOV2 severity phenotypes according to the infection’s time-onset and duration of symptoms, and link early responders with the up-regulation of key IFN/ISGs during the early infection stages, providing evidence for their better clinical outcome; and (vi) highly performing and robust machine-learning classifier models, founded on a set of 52 key IFN/ISGs, to aid and support (a) the diagnosis of SCOV2 infection when contrasted with acute respiratory illness events caused from other viral/non-viral infections, and (b) prognosis of COVID-19 severity (e.g., severe/critical vs. mild-moderate, symptomatic vs. asymptomatic).

Our results are more competent when compared with the results reported in the original studies of the datasets used in our experiments. Furthermore, our findings are in accordance with the conclusions of several relevant studies. A central conclusion of our study is that the molecular ‘norm’ governing SCOV2 infection follows a two-stage progression profile characterized by the inhibition of IFN-I genes and the blocking of key antiviral ISGs during the early infection stages. This leaves a critical time-window for virus replication (41) that has been shown to correlate with the two-stage SCOV2 infection profile (148). In agreement, in the top 10 articles announced by “Nature News” for the science discoveries in 2020, there are studies that demonstrate a strong association between IFN-Is and COVID-19, with robust secretion of IFN-Is to be an essential factor for the suppression of SCOV2 replication (109). IFNs, especially IFN-Is and the induced ISGs, encode a variety of antiviral effects throughout the whole viral life-cycle (entry, uncoating, genome replication, particle assembly and egress), with delayed or dysregulated IFN-I responses to be linked with the attraction of other immune components at the later infection stages (neutrophils, monocytes, dendritic cells, and natural-killer/NK cells) that guide to the accumulation of monocytes-macrophages into SCOV2 infected lungs (28, 149). An additional putative cause for the inhibition of IFN-Is at the early stages of SCOV2 infection is attributed to the role of ACE2 as an IFN-I induced/stimulated gene (150). In addition, recent reports suggest that detection of IFN-Is gene expression is of great importance in order to assess the severity of COVID-19 (151). Such an early failure of fundamental innate immune and resistance mechanisms results to uncontrolled hyperactivation of inflammatory reactions at the later stages of the infection, with hyperinflammation leading to the so-called ‘cytokine storm’, and finally to severe pneumonia, lung failure, and multiple organ damage, with potentially fatal outcomes. Indeed, a number of recent reports clearly indicate that such an aberrant antiviral response is a major contributor to the severity of the disease (152), with robust but non-tolerated IFN-mediated responses to occur in severe cases (153). Well-defined studies suggest that targeting early post-entry life cycle events is a common mode of IFN-I/ISG action (154). In particular, IFITMs (a specific ISG family) are included in the list of genes of the core SCOV2 molecular fingerprints induced by our experiments, inhibit the life-cycle of various viruses (including Influenza A and H1N1, filoviruses such as Embola, and SCOV1 as well) at their early steps, by blocking entry or viral particle trafficking (115, 155–157). In addition, it is known that several ISGs (including, IFI6, IFI27, IRFs, MX1, OAS1 and RSAD2/Viperin, also in the core SCOV2 molecular fingerprints induced by our experiments) reduce the activity of replicons (self-amplifying recombinant RNA molecules acting as virus-like particles) in HCV (hepatitis-C virus) (158). It is also shown that, in contrast to human common-cold coronaviruses (HCoVs), SCOV1/2 and MERS-CoV induce reduced IFN-I responses (52). As for the prophylactic and therapeutic treatment of SCOV2 infection, ′Frontiers in Immunology′ devote a special topic with three relevant key publications (159–161). In the last publication the authors report 14 highly preserved IFN-I related genes that are directly linked to different host response profiles, namely: BST2, IFIT1/2/3, IFITM1, ISG15, MX1/2, OAS1/2/3/L, RSAD2, and STAT1, with all of them to belong in the union of genes that compose the two-stage SCOV2 molecular fingerprints found by our experiments as down-regulated during the early stage of the infection (section 3.1.1).

The above supports in a great extend the soundness of our findings and strengthens their validity. As for future research, of major concern in the biomedical research community is the development of new COVID-19 vaccines with better long-standing effectiveness and enhanced ability to contain transmission. As it is reported in a recent meta-analysis study (162), the effectiveness of vaccines were particularly lower for the omicron SCOV2 variant compared to earlier variants, with the booster doses that covered mainly the omicron variant to drop from 70% against infections and 89% against hospitalization, to 43% and 71%, respectively, four months or more after vaccination. In another recent study (163), it is clearly stated that vaccine effectiveness for all omicron infections remains sparse, with the authors to report that about four months after immunization, vaccine effectiveness decreased to about 26% and 35% for three and four vaccine doses, respectively. It is established that higher IFN levels occur in the lower respiratory tract of severe patients, with the inverse to hold for the milder cases (164), and in this respect, mucosal immunization present a promising direction for the development of the new vaccines (165). We believe that our findings may provide valuable hints and putative targets to aid relevant research and development activities. Of course, translational research work and wet-lab experiments are needed in order to validate, screen, deploy and bring our findings from the bench to the bed side.

## Data availability statement

Fifteen publicly available datasets were analyzed in this study. This data can be found here: GSE151513: https://www.ncbi.nlm.nih.gov/geo/query/acc.cgi?acc=GSE151513 GSE158930: https://www.ncbi.nlm.nih.gov/geo/query/acc.cgi?acc=GSE158930 GSE33267: https://www.ncbi.nlm.nih.gov/geo/query/acc.cgi?acc=GSE33267 GSE148729: https://www.ncbi.nlm.nih.gov/geo/query/acc.cgi?acc=GSE148729 GSE47960: https://www.ncbi.nlm.nih.gov/geo/query/acc.cgi?acc=GSE47960 GSE152075: https://www.ncbi.nlm.nih.gov/geo/query/acc.cgi?acc=GSE152075 GSE156063: https://www.ncbi.nlm.nih.gov/geo/query/acc.cgi?acc=GSE156063 GSE166190: https://www.ncbi.nlm.nih.gov/geo/query/acc.cgi?acc=GSE166190 GSE161731: https://www.ncbi.nlm.nih.gov/geo/query/acc.cgi?acc=GSE161731 GSE152418: https://www.ncbi.nlm.nih.gov/geo/query/acc.cgi?acc=GSE152418 GSE178967: https://www.ncbi.nlm.nih.gov/geo/query/acc.cgi?acc=GSE178967 GSE172114: https://www.ncbi.nlm.nih.gov/geo/query/acc.cgi?acc=GSE172114 GSE177477: https://www.ncbi.nlm.nih.gov/geo/query/acc.cgi?acc=GSE177477 GSE188678: https://www.ncbi.nlm.nih.gov/geo/query/acc.cgi?acc=GSE188678 GSE163151: https://www.ncbi.nlm.nih.gov/geo/query/acc.cgi?acc=GSE163151 GSE156063: https://www.ncbi.nlm.nih.gov/geo/query/acc.cgi?acc=GSE156063.

## Author contributions

GP and PG contributed equally to this work. AK contributed to the design of the experiments and editing of the final manuscript. All authors contributed to the article and approved the submitted version.

## Footnotes

1
news.emory.edu/stories/2021/ 01/coronavirus_endemic_future/index.html

2 In the original publication it is reported that DESeq2’s ′median of ratios′ normalization method was used where, counts are divided by sample-specific size factors determined by median ratio of gene counts relative to geometric mean per gene (59).

3 HUGO Gene Nomenclature Committee, www.genenames.org

4 g:profiler/Gene ID conversion, https://biit.cs.ut.ee/gprofiler/convert

5 The bracketed entry indicates that the paragraph refers to the conducted experiment and to the respective results produced with a specific dataset (in this case GSE151513); the datasets used for respective tasks are presented in **Table 1**.

6 The average of the normalized gene’s expression values over the respective samples; see footnote in page 7 for the normalization method used for the GSE151513 dataset.

7 Genes in BMTs are listed in ′btm_annotation_table.xls′ file acquired from https://www.ncbi.nlm.nih.gov/ pmc/articles/ PMC3946932/bin/ NIHMS540680-supplement-26.zip

8
https://www.geeksforgeeks.org/binning-in-data-mining/
