# Supplementary material for: The two-stage molecular scenery of SARS-CoV-2 infection with implications to disease severity: An in-silico quest
Source: Front Immunol. 2023 Nov 21;14:1251067. doi: 10.3389/fimmu.2023.1251067 (PMC10699200; doi:10.3389/fimmu.2023.1251067)
Supplement: Supplementary file 1 [file DataSheet_1.zip › Supplement.pdf]

Supplementary Material

The two-stage molecular scenery of SARS-CoV-2 infection with implications to disease severity: An in-silico quest

George Potamias[1*†], Polymnia Gkoublia[1,2†], Alexandros Kanterakis[1]

[1]Computational Biomedicine Laboratory (CBML), Institute of Computer Science, Foundation for Research and Technology-Hellas (FORTH), Heraklion, Crete, Greece

[2]Graduate Bioinformatics Program, School of Medicine, University of Crete, Heraklion, Crete, Greece

*** Correspondence:** George Potamias**,** potamias@ics.forth.gr

1 Supplementary Data

2 Supplementary Figures and Tables

Fourteen figures: Supplementary Figure **1**, Supplementary Figure **2**, Supplementary Figure **3**, Supplementary Figure **4**, Supplementary Figure **5**, Supplementary Figure **6**, Supplementary Figure **7**, Supplementary Figure **8**, Supplementary Figure **9**, Supplementary Figure **10**, Supplementary Figure **11**, Supplementary Figure **12**, Supplementary Figure **13**, Supplementary Figure **14**.

One Table: Supplementary Table **1**.

2.1 Supplementary Figures

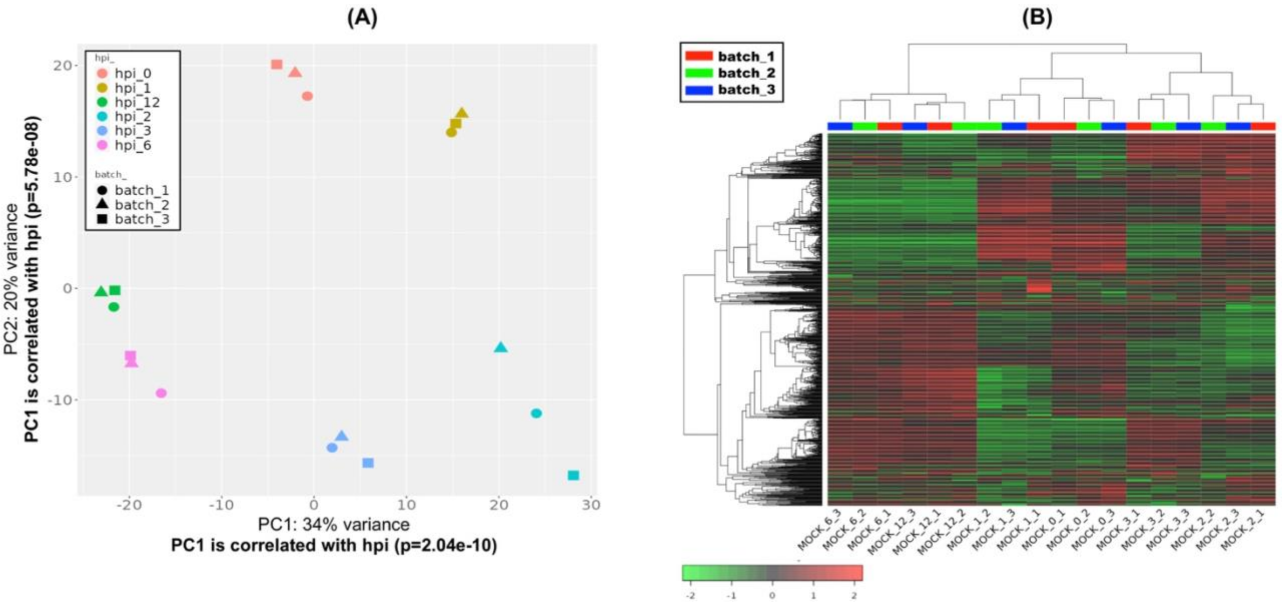

Supplementary Figure 1. (A) PCA plot of mock-treated samples; samples are grouped according to the hpi (hours post infection) variable and not according to the samples’ replicates/batches variable. (B) Heatmap of mock-treated samples which also indicates that clustering of samples is done according to the hpi variable.

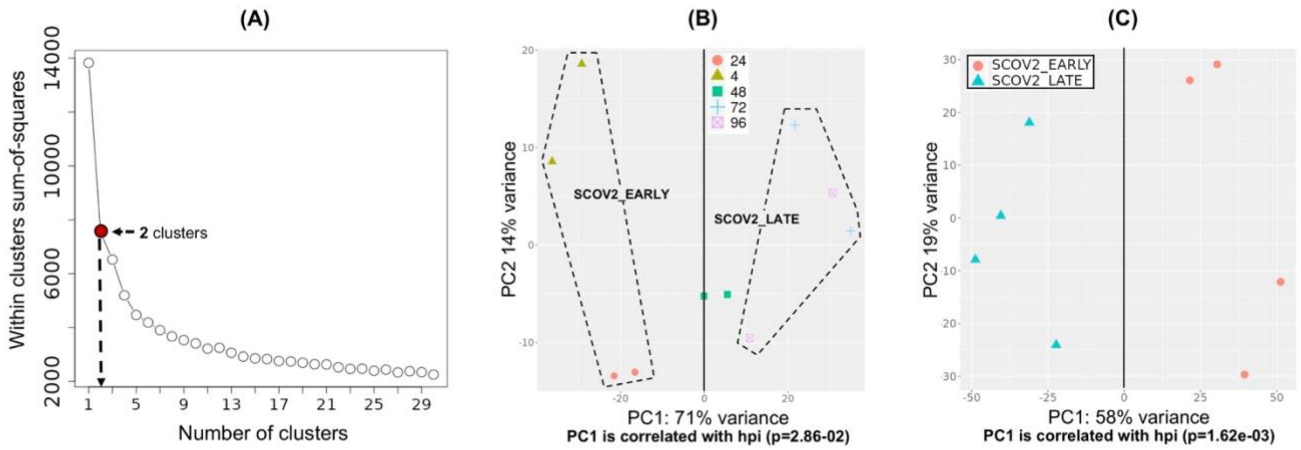

Supplementary Figure 2. Designation of infection stages. [GSE158930]. **(A)** Selection of the optimal number of clusters with the Elbow method for k-means clustering. **(B)** Designation of infection stages based on the inspection of samples' PCA plot on the cluster of genes found enriched in immune/defense GO-biological-processes; samples in hpis 4, 24 and 72, 96 designate the EARLY and LATE SCOV2 infection stages, respectively. **(C)** PCA plot of the samples (on all input genes) when assigned to the respective SCOV2_EARLY and SCOV2_LATE infection stage classes; the line corresponds to the PC with the largest (explained) variance, here PC1 (58%) is strongly correlated with hpi (p=1.62e-03).

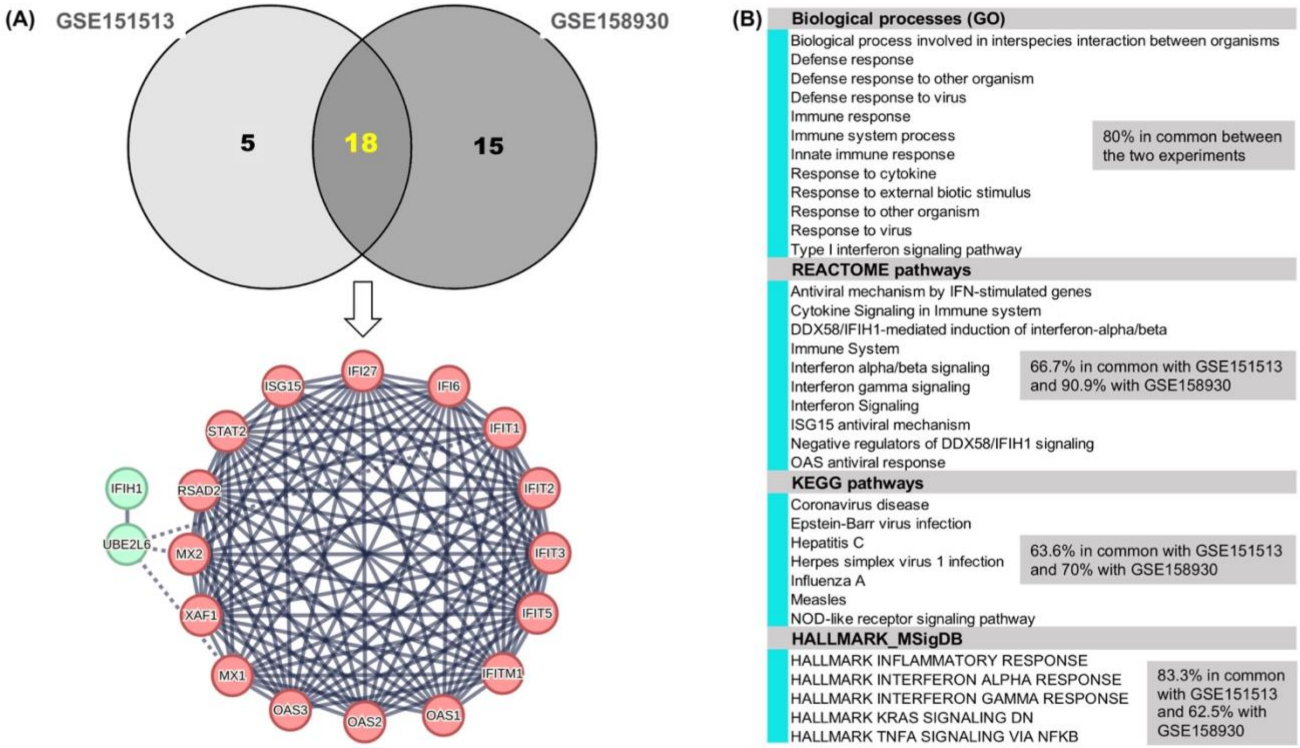

Supplementary Figure 3. [GSE151513, GSE158930]. **(A)** The 18 genes being down-regulated in the early SCOV2 stage that are shared between the two two-stage SCOV2 core molecular fingerprints induced by the experiments with datasets GSE151513 and GSE158930. **(B)** The respective shared enriched entries (GO-biological processes, KEGG/REACTOME pathways and hallmark signatures).

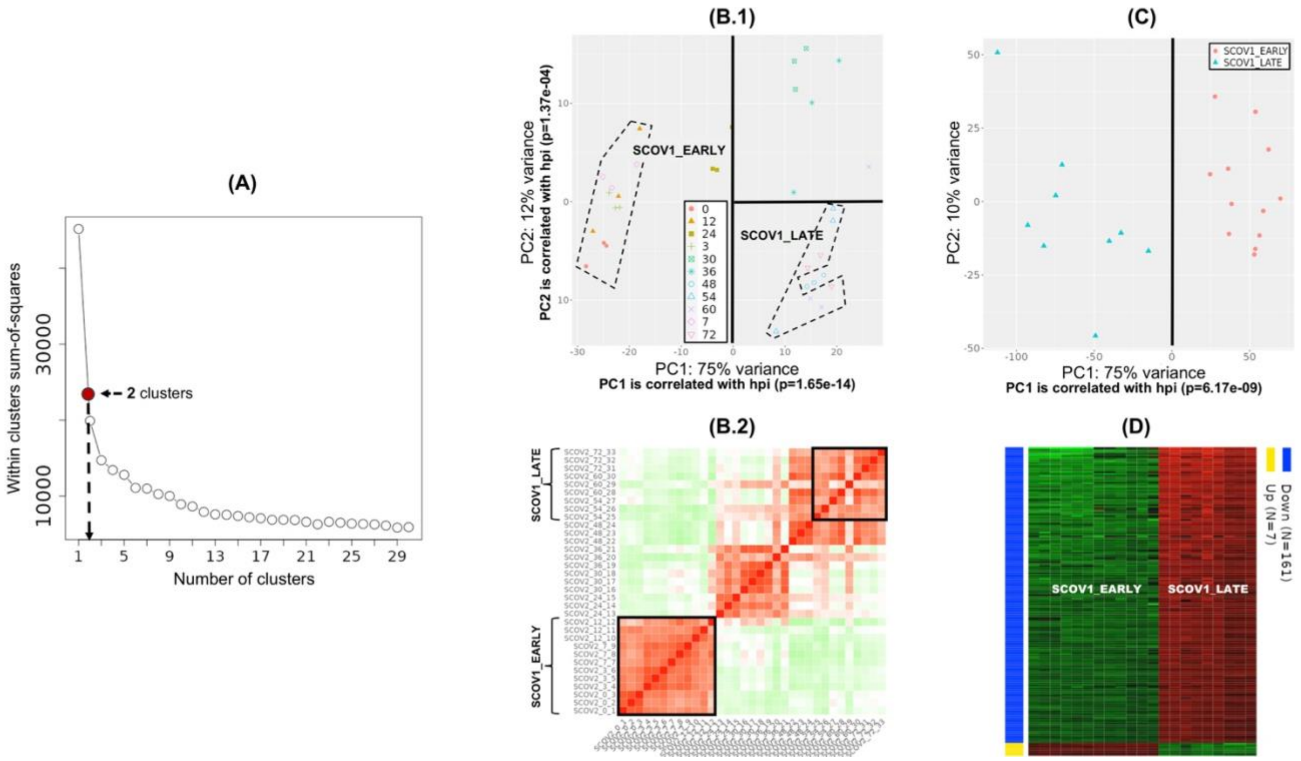

Supplementary Figure 4. Designation of infection stages & induction of DEGs. [GSE33267]. **(A)** Selection of the optimal number of clusters with the Elbow method for k-means clustering. **(B.1)** Designation of infection stages based on the inspection of samples' PCA plot on the cluster of genes found enriched in immune/defense GO-biological-processes; samples in hpis 0, 3, 7 and 12 designate the EARLY, and samples in hpis 54, 60 and 72 the LATE SCOV1 infection stage, respectively. **(B.2)** Correlation matrix (on the upper 75% most variable genes) of samples' gene-expression values, with indication of the samples assigned to the respective EARLY/LATE infection stages. **(C)** PCA plot of the samples when assigned to the respective SCOV1_EARLY and SCOV1_LATE infection stage classes; the line corresponds to the PC with the largest (explained) variance, here PC1 (75%) is strongly correlated with the hpi variable (p=6.17e-09). **(D)** Heatmap of samples' gene-expression values on the 168 induced DEGs, 7 up- and 161 down-regulated in the SCOV1_EARLY infection stage.

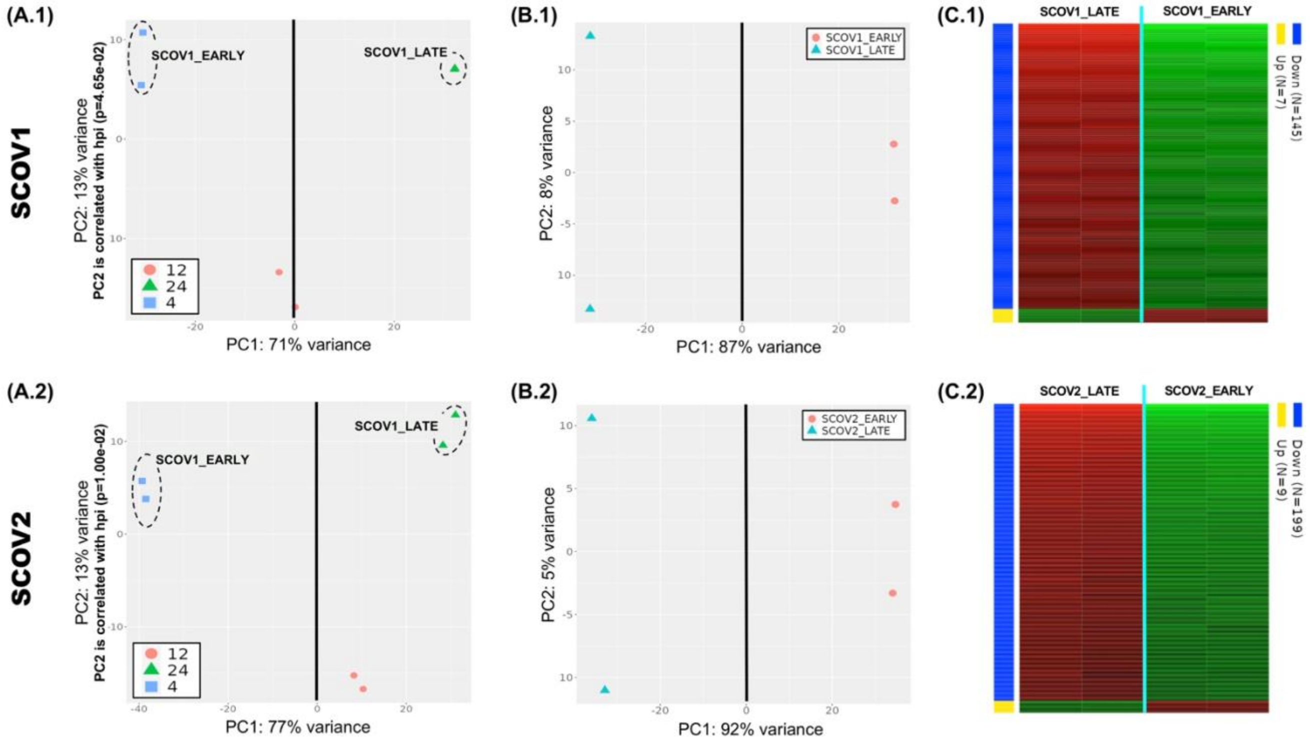

Supplementary Figure 5. Designation of infection stages & induction of DEGs. [GSE148729]. **(A.1)** Designation of infection stages for the GSE148729/SCOV1 infected cases based on the inspection of samples' PCA plot on the cluster of genes found enriched in immune/defense GO-biological-processes; samples in hpi 4 designate the EARLY, and samples in hpi 24 the LATE SCOV1 infection stage, respectively. **(B.1)** PCA plot of the samples when assigned to the SCOV1_EARLY/LATE classes. **(C.1)** Heatmap of samples' gene-expression values on the 168 induced DEGs (7 up- and 161 down-regulated in the EARLY SCOV1 infection stage). **(A.2)**, **(B.2)** and **(C.2)** shows the corresponding figures for the GSE148729/SCOV2 infected cases.

(A)

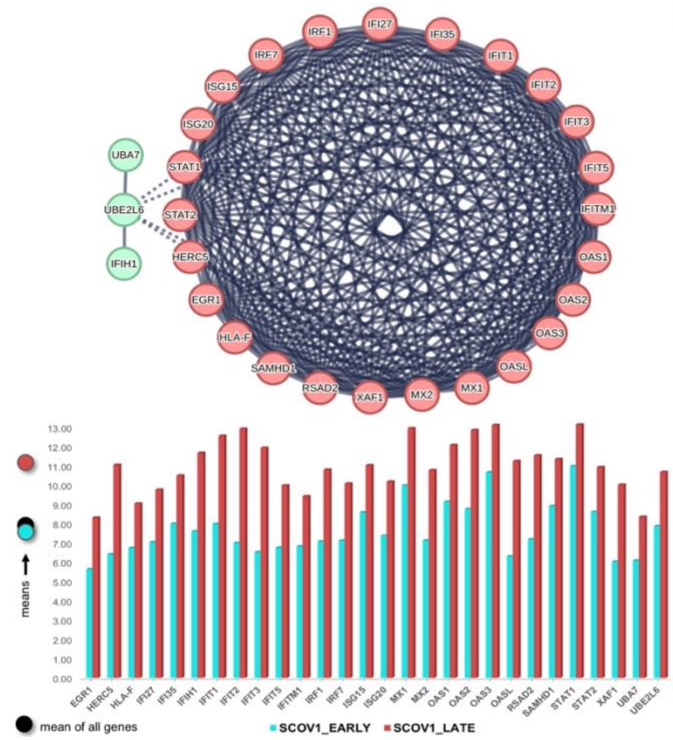

(C) Down-regulated in SCOV1_EARLY stage // Up-regulated in SCOV1_LATE stage

Biological processes (GO)	adj.Pval	#Genes
Response to biotic stimulus	6.00E-45	74
Response to other organism	6.00E-45	73
Biological process involved in interspecies interaction between organisms	1.30E-42	75
Defense response to other organism	2.30E-40	62
Innate immune response	5.60E-40	58
Response to cytokine	1.20E-36	64
Defense response	1.30E-36	67
Cytokine-mediated signaling pathway	8.20E-34	52
Defense response to virus	2.40E-33	37
Cellular response to cytokine stimulus	2.00E-32	58
Response to virus	3.70E-32	40
Immune response	3.80E-32	73
Type I interferon signaling pathway	9.30E-32	27
Cellular response to type I interferon	9.30E-32	27
Response to external stimulus	1.00E-31	78
**REACTOME pathways**	**adj.Pval**	**#Genes**
Interferon Signaling	1.80E-33	35
Cytokine Signaling in Immune system	1.70E-28	49
Interferon alpha/beta signaling	1.70E-28	22
Immune System	8.40E-22	62
Interferon gamma signaling	5.50E-14	14
Antiviral mechanism by IFN-stimulated genes	4.30E-12	15
Negative regulators of DDX58/IFIH1 signaling	1.00E-08	9
ISG15 antiviral mechanism	6.50E-08	11
DDX58/IFIH1-mediated induction of interferon-alpha/beta	8.30E-08	10
OAS antiviral response	1.20E-06	5
Activation of anterior HOX genes in hindbrain development during early embryogenesis	1.30E-04	8
Activation of HOX genes during differentiation	1.30E-04	8
Signaling by Interleukins	3.30E-04	16
Senescence-Associated Secretory Phenotype SASP	4.80E-04	7
Transcriptional regulation of granulopoiesis	4.90E-04	6
**KEGG pathways**	**adj.Pval**	**#Genes**
Measles	7.00E-10	14
Influenza A	7.00E-10	15
NOD-like receptor signaling pathway	1.10E-09	15
Epstein-Barr virus infection	1.10E-07	14
TNF signaling pathway	2.30E-07	11
Hepatitis C	5.30E-07	12
Coronavirus disease	1.50E-06	13
NF-kappa B signaling pathway	2.90E-04	7
RIG-I-like receptor signaling pathway	2.90E-04	6
Hepatitis B	2.90E-04	9
Systemic lupus erythematosus	3.70E-04	6
Herpes simplex virus 1 infection	4.90E-04	14
Viral protein interaction with cytokine and cytokine receptor	5.10E-04	4
Toll-like receptor signaling pathway	5.10E-04	6
Transcriptional misregulation in cancer	5.10E-04	8
**HALLMARK_MSigDB**	**adj.Pval**	**#Genes**
HALLMARK INTERFERON GAMMA RESPONSE	4.40E-56	47
HALLMARK INTERFERON ALPHA RESPONSE	1.10E-46	35
HALLMARK TNFA SIGNALING VIA NFKB	3.80E-22	26
HALLMARK INFLAMMATORY RESPONSE	7.80E-07	10
HALLMARK APOPTOSIS	1.10E-04	9
HALLMARK UV RESPONSE UP	5.10E-04	8
HALLMARK IL6 JAK STAT3 SIGNALING	6.60E-04	5
HALLMARK HYPOXIA	8.50E-04	8
HALLMARK KRAS SIGNALING UP	4.20E-03	6

Supplementary Figure 6. [GSE148729/SCOV1]. **(A)** Network of the 28 coherently clustered genes that compose the two-stage SCOV1 core molecular fingerprint for GSE148729/SCOV1 dataset, found as down-regulated in the EARLY SCOV1 stage. **(B)** Expression levels of the 28 genes that contrast between the respective EARLY/LATE SCOV1 stages. **(C)** Enriched entries found as down-regulated in the EARLY (up-regulated in the LATE) SCOV1 infection stage (notice the down-regulation of KEGG COVID-19 pathway indicated with blue color).

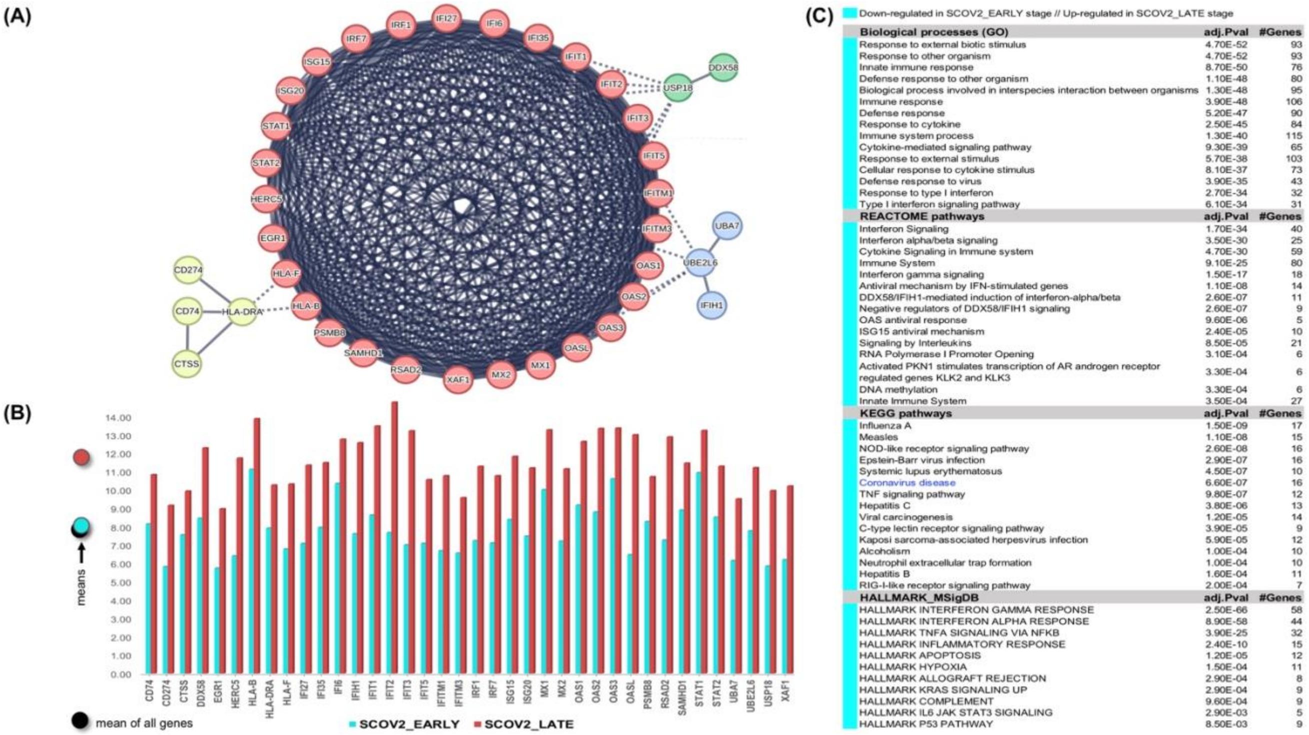

Supplementary Figure 7. [GSE148729/SCOV2]. **(A)** Network of the 38 coherently clustered genes that consist the two-stage SCOV2 core molecular fingerprint for GSE148729/SCOV2 dataset, and were found as down-regulated in the EARLY SCOV2 stage. **(B)** Expression levels of the 38 genes that contrast between the respective EARLY/LATE SCOV2 stages. **(C)** Enriched entries found as down-regulated in the EARLY (up-regulated in the LATE) SCOV2 infection stage (notice the down-regulation of KEGG COVID-19 pathway indicated with blue color).

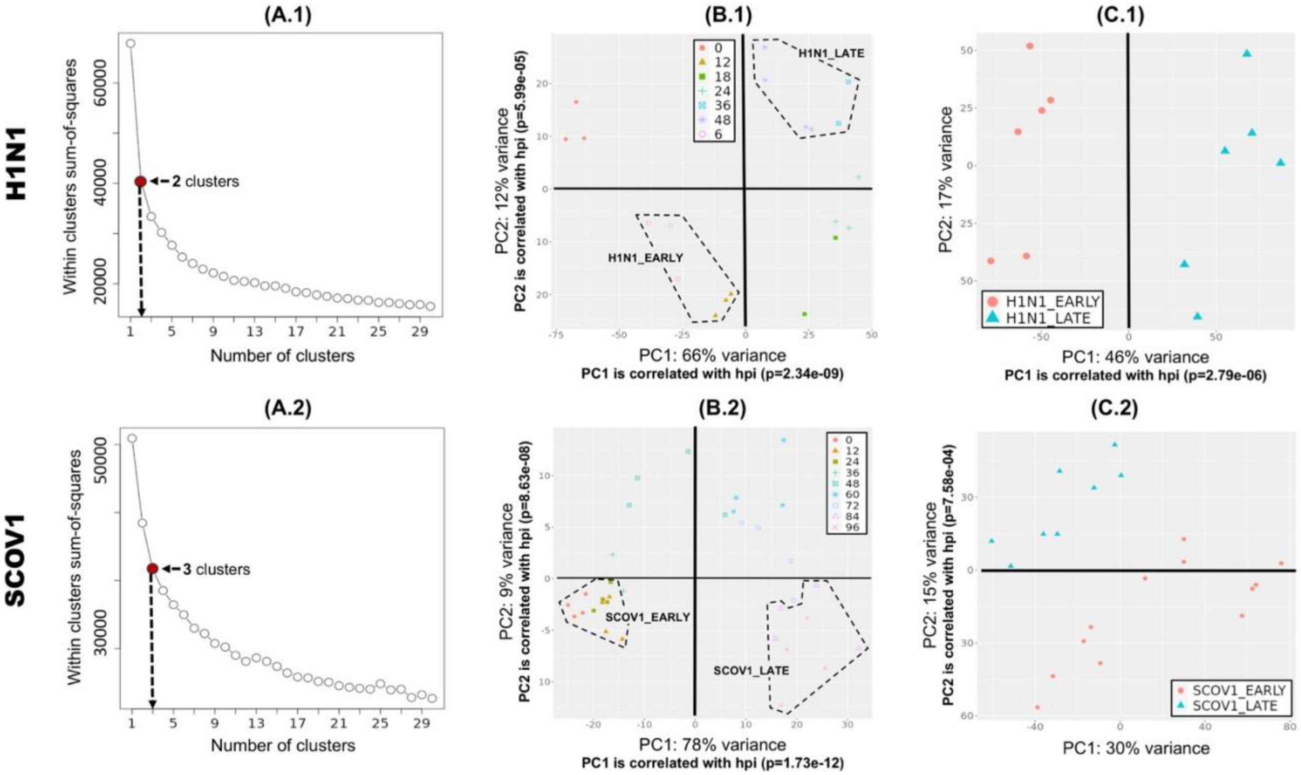

Supplementary Figure 8. Designation of infection stages. [GSE47960]. **(A.1)** Selection of the optimal number of clusters with the Elbow method for k-means clustering. **(B.1)** Designation of infection stages based on the inspection of the respective samples' PCA plot on the cluster of genes found enriched in immune/defense biological-processes; samples in hpis 6 and 12 designate the EARLY H1N1 infection stage. **(C.1)** PCA plot of the samples when assigned to the respective H1N1_EARLY/LATE infection stages; the line corresponds to the PC with the largest (explained) variance, here PC1 (46%) is strongly correlated with hpi (p=2.79e-06). **(A.2)**, **(B.2)** and **(C.2)** shows the corresponding figures for the GSE47960/SCOV1 infected cases.

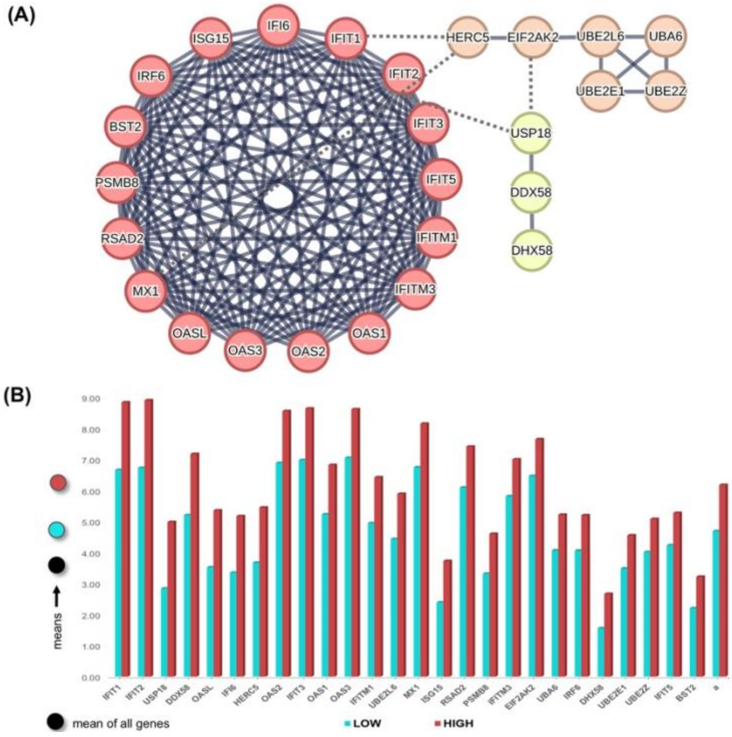

(C) Down-regulated in LOW viral load cases // Up-regulated in HIGH viral load cases

Biological processes (GO)	adj.Pval	#Genes
Response to virus	1.50E-18	42
Defense response to virus	1.50E-18	37
Response to external biotic stimulus	6.10E-17	76
Response to other organism	6.10E-17	76
Defense response	2.10E-16	78
Defense response to other organism	2.10E-16	64
Biological process involved in interspecies interaction between organisms	3.20E-15	79
Innate immune response	3.20E-15	56
Response to external stimulus	4.10E-12	95
Type I interferon signaling pathway	8.20E-12	19
Cellular response to type I interferon	8.20E-12	19
Response to cytokine	1.70E-11	62
Negative regulation of viral process	1.50E-10	19
Negative regulation of viral genome replication	6.00E-10	15
Immune response	5.00E-09	81
**REACTOME pathways**	**adj.Pval**	**#Genes**
Interferon Signaling	1.20E-12	28
Interferon alpha/beta signaling	2.60E-11	16
Interferon gamma signaling	9.20E-06	12
Cytokine Signaling in Immune system	3.70E-05	38
Antiviral mechanism by IFN-stimulated genes	3.70E-05	13
OAS antiviral response	2.20E-04	5
Immune System	2.50E-03	62
Antigen processingUbiquitination & Proteasome degradation	4.80E-03	18
ISG15 antiviral mechanism	7.80E-03	9
**KEGG pathways**	**adj.Pval**	**#Genes**
Influenza A	1.50E-05	13
Hepatitis C	1.50E-04	11
Cytosolic DNA-sensing pathway	3.10E-03	6
Measles	6.10E-03	8
Coronavirus disease	9.20E-03	10
Necroptosis	1.00E-02	8
**HALLMARK_MSigDB**	**adj.Pval**	**#Genes**
HALLMARK INTERFERON ALPHA RESPONSE	1.30E-57	44
HALLMARK INTERFERON GAMMA RESPONSE	1.30E-56	54
HALLMARK INFLAMMATORY RESPONSE	1.90E-06	14
HALLMARK COMPLEMENT	8.70E-06	13
HALLMARK TNFA SIGNALING VIA NFKB	3.80E-03	9
HALLMARK APOPTOSIS	3.80E-03	8
HALLMARK MTORC1 SIGNALING	3.80E-03	9
HALLMARK ANDROGEN RESPONSE	5.50E-03	6
HALLMARK CHOLESTEROL HOMEOSTASIS	7.20E-03	5

Supplementary Figure 9. [GSE152075]. **(A)** Network of the 26 coherently clustered genes, down-regulated in the low viral-load cases, that consist the SCOV2 core molecular fingerprint for low viral-load cases for GSE152075. **(B)** Expression levels of the 26 genes that contrast between the low/high viral-load cases. **(C)** Enriched entries found as down-regulated in the low viral-load (up-regulated in the high viral-load) SCOV2 infected cases (notice the down-regulation of KEGG COVID-19 pathway indicated with blue color).

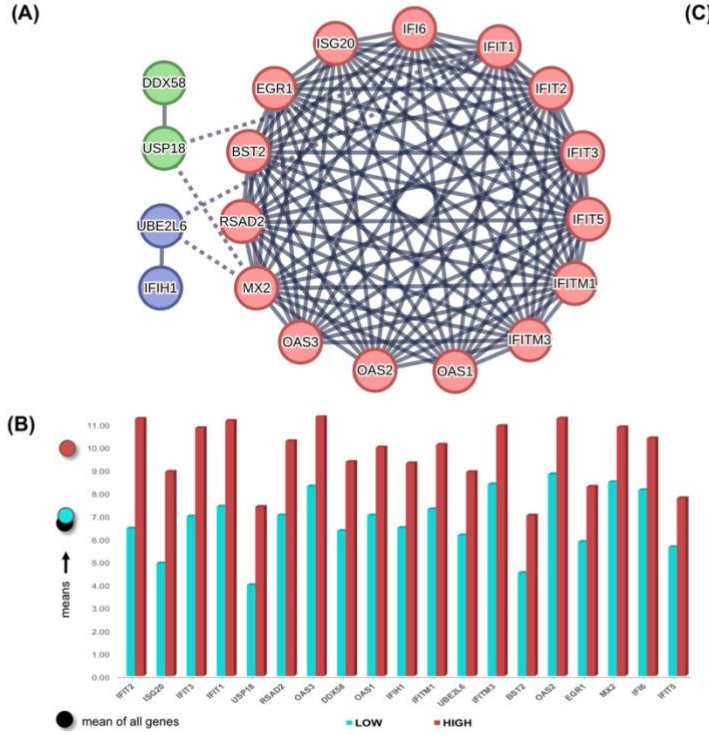

(C) Down-regulated in LOW viral load cases // Up-regulated in HIGH viral load cases

Biological processes (GO)	adj.Pval	#Genes
Defense response to virus	7.80E-17	21
Response to type I interferon	3.90E-16	17
Defense response	1.90E-15	34
Response to virus	1.90E-15	22
Type I interferon signaling pathway	2.20E-15	16
Cellular response to type I interferon	2.20E-15	16
Defense response to other organism	2.90E-15	30
Response to external biotic stimulus	3.50E-15	33
Response to other organism	3.50E-15	33
Response to cytokine	4.60E-15	32
Innate immune response	5.50E-15	28
Biological process involved in interspecies interaction between organisms	3.30E-13	33
Response to external stimulus	4.00E-13	38
Negative regulation of viral genome replication	2.10E-12	11
Immune response	1.70E-11	35
**REACTOME pathways**	**adj.Pval**	**#Genes**
Interferon Signaling	3.40E-17	21
Interferon alpha/beta signaling	3.40E-17	15
Cytokine Signaling in Immune system	9.50E-15	28
Immune System	3.30E-08	31
Antiviral mechanism by IFN-stimulated genes	2.70E-05	8
OAS antiviral response	2.80E-05	4
Interferon gamma signaling	2.90E-05	7
ISG15 antiviral mechanism	9.50E-03	5
**KEGG pathways**	**adj.Pval**	**#Genes**
Measles	2.40E-04	8
NOD-like receptor signaling pathway	4.00E-04	8
Influenza A	4.00E-04	8
Hepatitis C	1.60E-03	7
Coronavirus disease	1.60E-03	9
**HALLMARK_MSigDB**	**adj.Pval**	**#Genes**
HALLMARK INTERFERON ALPHA RESPONSE	2.10E-30	25
HALLMARK INTERFERON GAMMA RESPONSE	2.10E-30	29
HALLMARK TNFA SIGNALING VIA NFKB	2.90E-06	10
HALLMARK HYPOXIA	2.00E-03	6
HALLMARK APOPTOSIS	2.30E-03	6
HALLMARK INFLAMMATORY RESPONSE	2.30E-03	5
HALLMARK P53 PATHWAY	2.70E-03	6

Supplementary Figure 10. [GSE156063]. **(A)** Network of the 19 coherently clustered genes, down-regulated in the low viral-load cases, and consist the SCOV2 core molecular fingerprint for low viral-load cases for GSE156063. **(B)** Expression levels of the 19 genes that contrast between the low/high viral-load cases. **(C)** Enriched entries (GO-biological processes, REACTOME/KEGG pathways and Hallmark MSigDB signatures) found as down-regulated in the low viral-load (up-regulated in the high viral-load) SCOV2 infected cases (notice the down-regulation of KEGG COVID-19 pathway indicated with blue color).

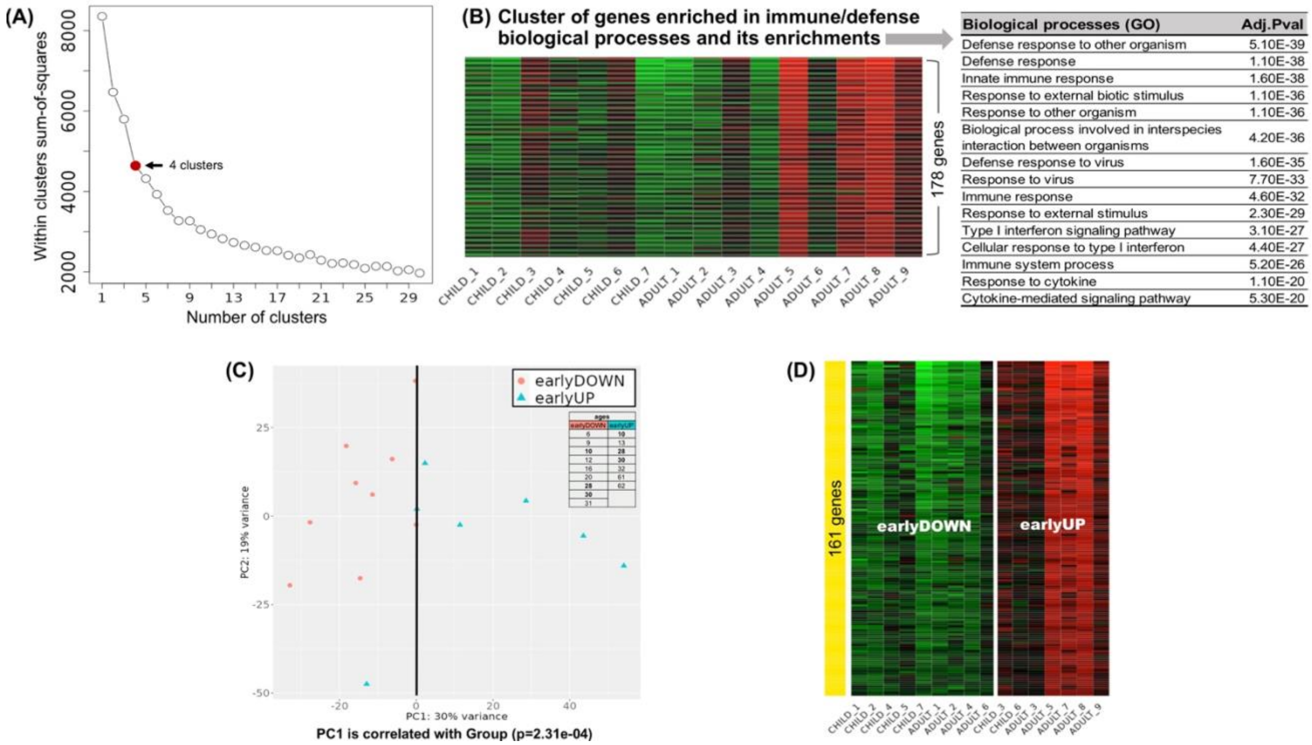

Supplementary Figure 11. Designation of early/late response groups. [GSE166190]. **(A)** Selection of the optimal number of clusters with the Elbow method for k-means clustering of the cases reporting early onset of symptoms (0-5 DPOS, n=16 cases, 9 adults and 7 children). **(B)** Heatmap of 178 genes that consist the cluster of genes found enriched in immune/defense GO-biological processes; their enrichments are also shown. **(C)** PCA plot of samples assigned (by inspecting the heatmap in (B)) to the earlyDOWN (n=9; 5 children/CHILD_1,2,4,5,7 and 4 adults/ADULT_1,2,4,6) and earlyUP (early responders, n=7; 2 children/CHILD_3,6 and 5 adults/ADULT_3,5,7,8,9) response groups, respectively; the ages of the cases assigned to each group are indicated. **(D)** Heatmap of samples' gene-expression values on the 161 induced DEGs being upregulated in the earlyUP response group; the separation between the earlyUP/DOWN samples is indicated (white vertical line).

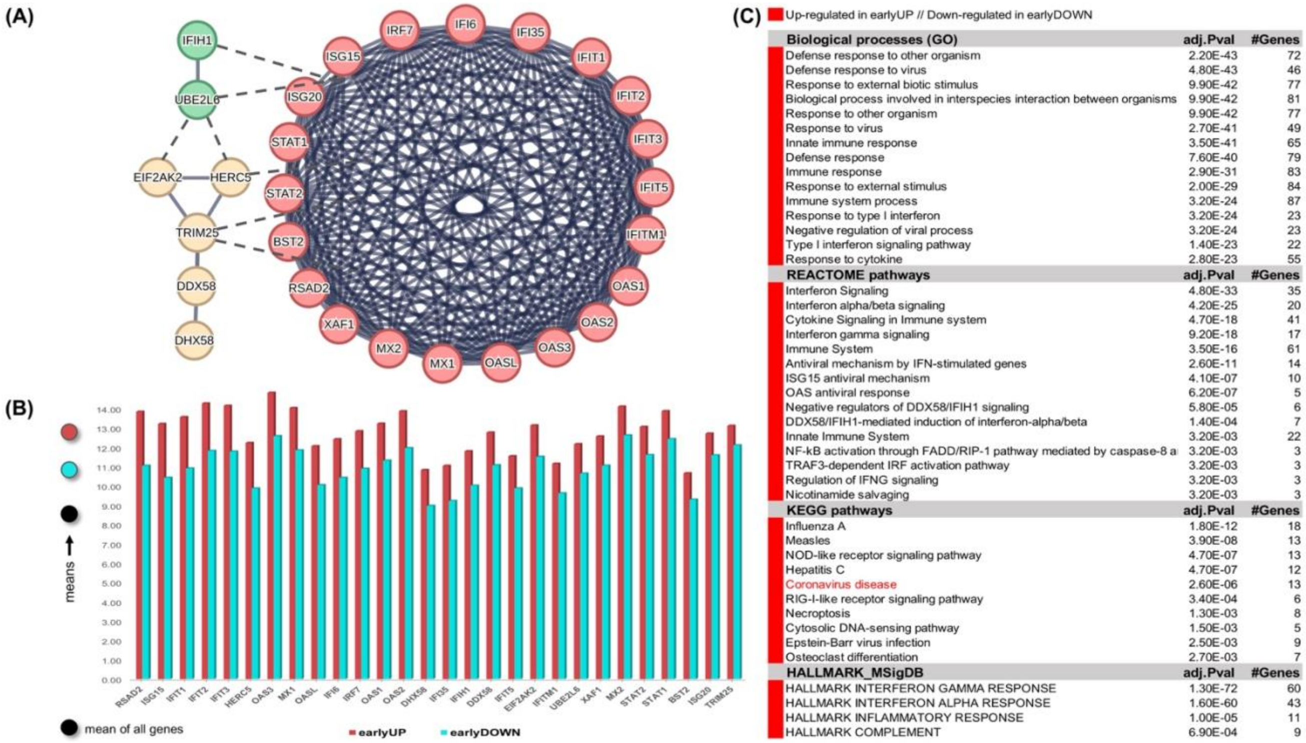

Supplementary Figure 12. [GSE166190, early responders]. **(A)** Network of the 28 coherently clustered genes being up-regulated in the earlyU group and consist the SCOV2 core molecular fingerprint for early responders. **(B)** Expression levels of the 28 genes that contrast between the earlyUP/earlyDOWN groups. **(C)** Enriched entries found up-regulated in the earlyUP group (down-regulated in the earlyDOWN group); notice the up-regulation of KEGG COVID-19 pathway indicated with red color.

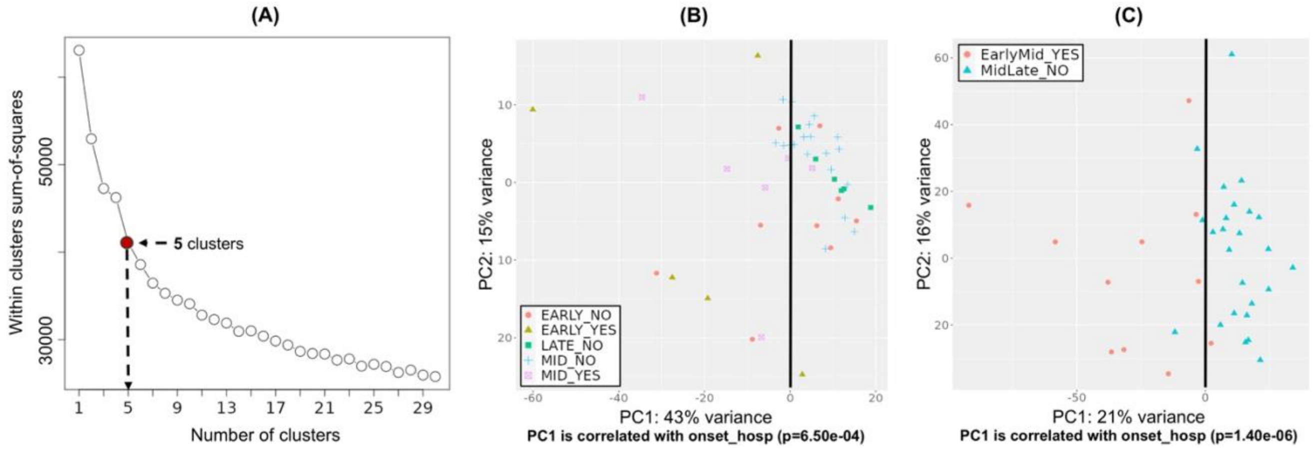

Supplementary Figure 13. Designation of early/late response groups. [GSE161731]. **(A)** Selection of the optimal number of clusters with the Elbow method for k-means clustering. **(B)** PCA plot of samples assigned to EARLY_NO/YES, LATE_NO and MID_YES/NO LATE_NO/YES onset_hosp classes; the line corresponds to the PC with the largest (explained) variance, here PC1 (43%) is correlated with the onset_hosp variable (p=6.50e-04). **(C)** PCA plot of the samples, on all genes, when assigned to the EarlyMid_YES/MidLate_NO groups; the line corresponds to the PC with the largest (explained) variance, here PC1 (21%) is correlated with the onset_hosp variable (p=1.4e-06).

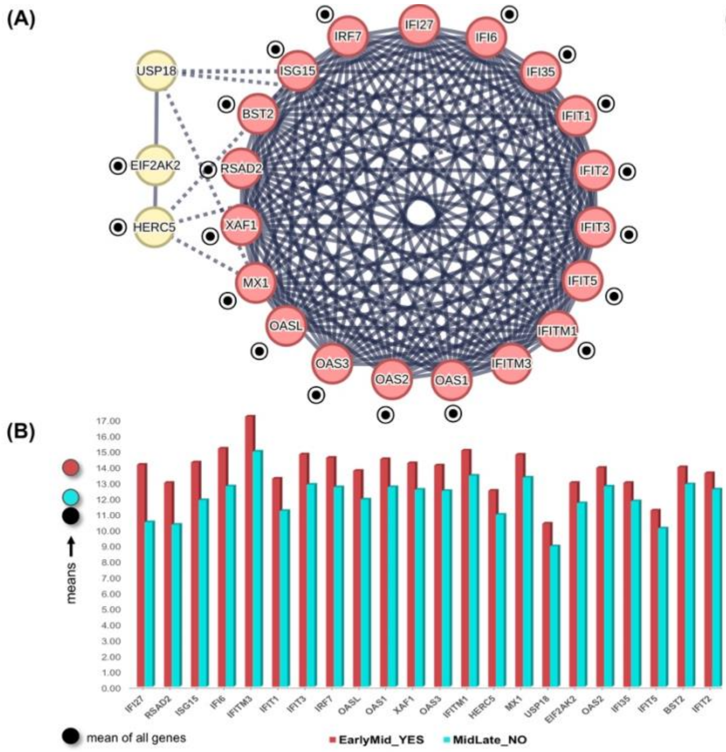

(C) Up-regulated in EarlyMid_YES stage // Down-regulated in MidLate_NO

Biological processes (GO)	adj.Pval	#Genes
Immune response	1.70E-29	95
Biological process involved in interspecies interaction between organisms	1.70E-29	79
Defense response	9.00E-29	78
Defense response to other organism	9.00E-29	66
Response to external biotic stimulus	2.20E-28	73
Response to other organism	2.20E-28	73
Innate immune response	4.70E-26	58
Response to external stimulus	1.60E-24	90
Immune system process	2.10E-24	102
Defense response to virus	2.50E-19	31
Type I interferon signaling pathway	3.60E-19	21
Cellular response to type I interferon	4.70E-19	21
Negative regulation of viral genome replication	5.90E-17	17
Response to virus	8.20E-17	32
Cytokine-mediated signaling pathway	1.10E-15	43
**REACTOME pathways**	**adj.Pval**	**#Genes**
Immune System	1.10E-20	79
Interferon alpha/beta signaling	8.90E-19	18
Interferon Signaling	1.40E-16	25
Cytokine Signaling in Immune system	1.60E-10	36
Innate Immune System	1.40E-09	41
Neutrophil degranulation	1.40E-09	29
Interferon gamma signaling	9.00E-07	10
Antiviral mechanism by IFN-stimulated genes	2.00E-05	10
Classical antibody-mediated complement activation	1.10E-04	3
Regulation of Complement cascade	1.80E-04	5
OAS antiviral response	1.80E-04	4
Complement cascade	2.70E-04	5
Creation of C4 and C2 activators	2.70E-04	3
Metal sequestration by antimicrobial proteins	2.70E-04	3
G1/S-Specific Transcription	6.00E-04	5
**KEGG pathways**	**adj.Pval**	**#Genes**
Pyrimidine metabolism	2.40E-04	7
Staphylococcus aureus infection	2.40E-04	7
Coronavirus disease	2.40E-04	13
Hepatitis C	4.10E-04	10
Drug metabolism	6.50E-04	6
Influenza A	6.50E-04	10
Complement and coagulation cascades	9.60E-04	5
Systemic lupus erythematosus	3.80E-03	6
**HALLMARK_MSigDB**	**adj.Pval**	**#Genes**
HALLMARK INTERFERON GAMMA RESPONSE	4.80E-31	38
HALLMARK INTERFERON ALPHA RESPONSE	2.00E-25	26
HALLMARK INFLAMMATORY RESPONSE	3.50E-06	13
HALLMARK G2M CHECKPOINT	4.70E-03	10
HALLMARK E2F TARGETS	5.00E-03	10
HALLMARK IL2 STAT5 SIGNALING	6.30E-03	9

Supplementary Figure 14. [GSE161732, early responders]. **(A)** Network of the 22 coherently clustered genes, up-regulated in the EarlyMid_YES group, that consist the SCOV2 core molecular fingerprint for early responders. **(B)** Expression levels of the 22 genes that contrast between the respective EarlyMid_YES (early responders)/MidLate_NO cases; circled black-dots indicate the 19 genes being shared between the 22 genes and the 28 fingerprint genes for the earlyUP group induced from the previous experiment with GSE166190 dataset. **(C)** Enriched entries found up-regulated in the EarlyMid_YES group (down-regulated in the MidLate_NO group); notice the up-regulation of KEGG COVID-19 pathway indicated with red color.

2.2 Supplementary Tables

Supplementary Table 1. Summary of the set-up followed for each of the performed experiments

<table>
<tr><th>Task</th><th>Dataset</th><th>P;T;G / #[1]</th><th>(Gene Filtering)
MethodGF /
#RetainedG[2]</th><th>(Normalization-
Transformation)
Norm./MethodN[3]</th><th>stage-pheno / hpis / #[4]</th><th>FDR</th><th>FC</th><th>U / D[5]</th><th>#Core[6]</th></tr>
<tr><td rowspan="2">1</td><td>GSE151513</td><td>T / 15,761</td><td>40% / 9,457</td><td>yes / DESeq2**</td><td>EARLY / 0,1,2 / 9
LATE / 12 / 3</td><td rowspan="12">0.05</td><td>2</td><td>79 / 53</td><td>23</td></tr>
<tr><td>GSE158930</td><td>T / 57,905</td><td>CPM<5 (all)[7] / 9,833</td><td>no / VST</td><td>EARLY / 4,24 / 4
LATE / 72,96 / 4</td><td>4</td><td>15 / 160</td><td>33</td></tr>
<tr><td rowspan="3">2</td><td>GSE33267</td><td>P / 33,631</td><td>40% / 10,353</td><td>yes / quantile-log2</td><td>EARLY / 0,3,7,12 / 12
LATE / 54,60,72 / 9</td><td>8</td><td>7 / 161</td><td>18</td></tr>
<tr><td>GSE148729 (SCOV1)</td><td rowspan="2">T / 40,648</td><td rowspan="2">CPM<5 (all) / 10,535
(Ensembl)</td><td rowspan="2">no / VST</td><td rowspan="2">EARLY / 4 / 2
LATE / 24 / 2</td><td rowspan="2">4</td><td>7 / 145</td><td>28</td></tr>
<tr><td>GSE148729 (SCOV2)</td><td>9 / 199</td><td>38</td></tr>
<tr><td rowspan="2">3</td><td>GSE47960 (H1N1)</td><td rowspan="2">P / 32,067</td><td rowspan="2">yes / 32,067</td><td rowspan="2">yes / quantile</td><td>EARLY / 6,12 / 6</td><td rowspan="2">4</td><td rowspan="2">191 / 2</td><td rowspan="2">29</td></tr>
<tr><td>GSE47960 (SCOV1)</td><td>EARLY / 0,12,24 / 12</td></tr>
<tr><td rowspan="2">4</td><td>GSE152075</td><td>G / 35,784</td><td>CPM<2 (59)[7] / 8,130</td><td>no / VST</td><td>LOW / na*** / 59
HIGH / na / 72</td><td>2</td><td>18 / 283</td><td>26</td></tr>
<tr><td>GSE156063</td><td>T / 15,979</td><td>CPM<5 (all) / 3,551</td><td>no / VST</td><td>LOW / na / 33
HIGH / na / 33</td><td>4</td><td>0 / 56</td><td>19</td></tr>
<tr><td rowspan="2">5</td><td>GSE166190</td><td>T / 58,825</td><td>CPM<3 (half)[7] / 13,027</td><td>no / VST</td><td>earlyUP / na / 7
earlyDOWN / na / 9</td><td>2</td><td>161 / 0</td><td>28</td></tr>
<tr><td>GSE161731</td><td>T / 60,675</td><td>CPM<5 (10%)[7] / 11,497</td><td>no / VST</td><td>EarlyMid_YES / na / 11
MidLate_NO / na / 25</td><td>2</td><td>250 / 61</td><td>22</td></tr>
</table>

[1]**P;T;G / #:** Probes; (Ensembl)Transcripts; Genes (HGNC) / umber of Ps, Ts or Gs
[2]**MethodGF / #RetainedG**: Gene Filtering method* / number of retained HGNC genes
*% CPM cutoff or % of low expressed genes discarded (based on their maximum value over all samples)
[3]**Norm. / MethodN**: Already normalized? (yes, no) / (if no) VST transformation
[4]**stage-pheno / hpis / #**: designated infection stage (e.g., early/late) name or phenotypic class name **/** hours post infection assigned to stage **/** number of samples assigned to the designated stage or to the designated phenotypic class
[5]**U/D**: induced Up-/Down-regulated genes
[6]**#Core**: number of genes included in the core molecular fingerprint (genes included in the STRING/MCL coherent clusters)
[7]**all/half**: over all samples/over half of the samples; **N/N%**: over Number of samples / over % of the samples

**DESeq2's ″median ratio method″
***na: not applicable
